# HawkesRank: Event-driven centrality for real-time importance ranking

**DOI:** 10.1093/pnasnexus/pgag292

**Published:** 2026-09-01

**Authors:** Didier Sornette, Yishan Luo, Sandro Claudio Lera

**Affiliations:** Institute of Risk Analysis, Prediction and Management, Southern University of Science and Technology, Shenzhen 518055, China; Institute of Risk Analysis, Prediction and Management, Southern University of Science and Technology, Shenzhen 518055, China; Warwick Business School, University of Warwick, Coventry CV4 7AL, United Kingdom; Institute of Risk Analysis, Prediction and Management, Southern University of Science and Technology, Shenzhen 518055, China

**Keywords:** dynamic centrality, Hawkes processes, network ranking, event-driven models, information diffusion

## Abstract

Quantifying influence in networks is important across science, economics, and public health, yet widely used centrality measures remain limited: they rely on static representations, heuristic network constructions, and purely endogenous notions of importance, while offering little semantic connection to observable activity. We show that influence can instead be interpreted through multivariate Hawkes point processes that model exogenous drivers and endogenous amplification (self- and cross-excitation). This yields a principled, empirically calibrated, and adaptive notion of importance grounded in observable event dynamics. Classical indices such as Katz centrality and PageRank emerge as stationary mean-field limits of this formulation, clarifying both their interpretation and their limitations. Unlike static averages, the resulting rankings are determined by instantaneous event intensities, allowing rankings to adapt to shocks while distinguishing endogenous amplification from exogenous activity. Using both simulations and empirical analysis of emotion dynamics in online communication platforms, we show that this event-driven formulation captures temporally evolving system activity more effectively than static centrality metrics.

Significance statement Centrality measures are widely used to quantify importance in networks, but they are usually defined on static graphs and assign influence through recursive network structure alone. As a result, they are poorly equipped to describe evolving systems in which importance is shaped both by external drivers and by endogenous feedback. Here, we show that influence can instead be formulated through self-exciting event dynamics, with classical measures such as Katz centrality and PageRank emerging as limiting cases. This event-based perspective gives centrality a direct connection to observable activity, yields rankings that adapt over time, and provides an interpretable decomposition of influence into exogenous and endogenous components.

## Introduction

We live in an era of information overload, where attention has become an increasingly scarce resource (1). To navigate this complexity, we rely on ranking systems to assess relevance and prioritize options. As a result, rankings have become ubiquitous in modern society, playing a central role in decision-making across domains. University and academic rankings shape the distribution of talent, rankings of stocks and financial instruments guide the allocation of capital, and website search rankings have been shown to influence election outcomes by swaying undecided voters (2). Rankings therefore strongly influence how attention, resources, and opportunities are allocated across modern socio-economic systems.

Many ranking systems are built upon network representations of interactions. Within these networks, centrality measures provide systematic tools for identifying the most important or influential nodes. Different metrics capture different structural aspects of connectivity. Among them, eigenvector-based measures, such as Katz centrality and PageRank, form a particularly influential class, emphasizing that relevance accrues through association with already influential nodes. Katz centrality captures this by summing over all walks in the network with an attenuation factor that discounts longer paths, while PageRank operationalizes a similar idea through a random-walk model that ranks web pages (3).

By translating connectivity into quantitative scores of influence, network-based and recursive ranking methods have found applications across domains, including the ranking of countries and products by economic complexity (4), financial institutions by systemic risk (5, 6), students by popularity (7), politicians by influence (8, 9), individuals in crime networks by criminal activity (10, 11), startups by expected success (12, 13), law firms by win rate (14), scientific papers and researchers by impact (15–17), and athletes by performance in sports analytics (18, 19). They have also been used to identify central neurons in brain networks (20, 21), to analyze protein interaction networks (22), and to assess species importance in ecological systems (23).

Despite their success, classical centrality measures suffer from at least five interdependent limitations. First, they inadequately capture exogenous (henceforth *exo*) drivers of relevance. Eigenvector-based centralities rely on a self-referential logic, assigning importance to nodes connected to other important nodes. Such endogenous (henceforth *endo*) formulations neglect activity generated independently of the network. Although Katz centrality includes an exo baseline term, it is typically assumed homogeneous or omitted altogether, limiting the ability to account for real-world drivers such as media exposure, advertising, policy shocks, or intrinsic appeal (24–26).

Second, standard centrality measures are static. They assume fixed network structures and immutable relevance scores, making them unresponsive to changing environments or new information (27, 28). In practice, rankings must adapt to shifting attention, shocks, and emergent trends, something current metrics can only achieve through repeated retraining. Even PageRank, though widely viewed as dynamic, is usually applied to static graphs and exhibits built-in temporal bias: earlier nodes are favored over newer entrants, entrenching outdated rankings and amplifying systemic feedback (26). Several temporal-network centrality measures have been proposed to address some of these limitations by incorporating time-dependent interactions, tie decay, or evolving connectivity patterns (29, 30). These approaches provide important extensions of static centrality concepts when temporally resolved dyadic interaction data are available. However, they typically remain based on evolving adjacency structures and prescribed temporal propagation rules, requiring time-stamped edge information or additional edge-construction procedures. By contrast, HawkesRank operates directly on event sequences through a generative point-process framework that jointly models endogenous excitation and exogenous activity without requiring an intermediate temporal-network reconstruction step.

Third, the network structures on which centrality measures operate are often constructed in an ad hoc manner. In many applications, the adjacency matrix is not directly observed but inferred from data through heuristic choices, such as temporal aggregation windows, thresholding rules, or normalization schemes. For example, interaction networks are frequently built by linking two events whenever they occur within a chosen time interval, or by projecting bipartite data onto one-mode networks. These procedures introduce multiple degrees of freedom that can substantially alter the resulting network and therefore the inferred rankings. As a result, centrality scores often depend as much on modeling assumptions as on the underlying data, limiting their statistical interpretability and robustness.

Fourth, classical centrality measures often lack semantic clarity: it is not well-defined what quantity is being ranked. Eigenvector-based scores are dimensionless and lack direct physical interpretation, which limits their comparability across contexts and their connection to observable outcomes. This weakens both the interpretability and empirical grounding of traditional rankings.

Fifth, existing methods conflate qualitatively different types of exo influence. External signals often blend slowly evolving intrinsic value with transient visibility surges from advertising, social amplification, or opportunistic exposure. When the former dominates, exogenous activity reflects fundamental importance; when the latter prevails, rankings may be distorted by ephemeral or strategic effects. This distinction is central to disciplines such as ethics (31, 32), information systems (33), finance (34), and development economics (35), yet remains unresolved: intrinsic value is often abstract, context-dependent, and unobservable. In many socio-economic systems, externally amplified visibility and baseline relevance may become difficult to disentangle, particularly when strategic signaling, platform amplification, or media exposure strongly influence observed activity.

Taken together, these limitations show that ranking in complex systems requires a framework capable of jointly addressing several intertwined challenges: disentangling endogenous amplification from heterogeneous exogenous drivers, accommodating temporal evolution and shocks, reducing dependence on arbitrary network constructions, and distinguishing persistent baseline activity from transient or strategically amplified visibility. To address these intertwined limitations, we formulate ranking as an event-driven process based on self-exciting multivariate Hawkes processes (SEMHP) (36–40). We refer to this formulation as *HawkesRank*. Within this framework, importance is interpreted as the conditional propensity of a node to generate or receive influence-mediated events given the observed event history. It explicitly disentangles endogenous amplification from heterogeneous exogenous drivers while accounting for the temporal evolution of influence. Previous applications of SEMHP have focused on modeling bursty dynamics for trend detection and popularity prediction, such as the viral spread of tweets (41, 42), online emotions (43), or the inference of hidden influence networks from social media interactions (44). Our contribution differs in that we repurpose this class of models as a general analytical tool for ranking, providing a statistically grounded framework for separating endogenous reinforcement from exogenous contributions. We show that, in the static limit, HawkesRank recovers Katz centrality. But unlike static centrality measures, HawkesRank explicitly decomposes activity into time-varying endogenous and exogenous components, where the exogenous term represents activity not explained by endogenous excitation within the specified model. This decomposition yields event-based rankings that adapt to memory effects, network reinforcement, and evolving external conditions.

## Results

### HawkesRank: a multivariate Hawkes process

We now formalize *HawkesRank* using the mathematics of SEMHP. SEMHP captures the idea that events can arise both from an exogenous background rate of activity and from cascades triggered by earlier events, and provides one of the simplest mathematically tractable extensions of Poisson point processes capable of capturing self-excitation, cross-excitation, and cascade dynamics.

Formally, we consider *M* distinct *event types*. In the network representation used by centrality metrics, these *M* objects correspond to the *M* nodes of the network. For each event type i=1,…,M, the SEMHP specifies an intensity function $\lambda_{i}(t)$ that describes the instantaneous rate at which events of type *i* are expected to occur at time *t*, that is, the probability of observing an event of type *i* in a short interval [t,t+dt) is equal to $\lambda_{i}(t)dt$. Importantly, $\lambda_{i}(t)$ is a conditional intensity process defined with respect to the observed event history. Once the realized event sequence and model parameters are specified, the corresponding intensity trajectory is fully determined conditional on that information. In the remainder of this article, we assume this intensity is given by


(1) λi(t)=μi(t)∑j=1M∑k:tkj<tni,jϕ(t−tkj)⏟exo+∑j=1M∑k:tkj<tni,jϕ(t−tkj)⏟endo

with more general versions of the SEMHP discussed in the Methods section below.

This expression consists of two components. The first term, the background, exogenous (exo) component $\mu_{i}(t)$, captures the idea that events may occur spontaneously due to external drivers or baseline activity not explained by endogenous excitation. For example, in the context of website visits, $\mu_{i}(t)$ reflects any factor that draws user attention to page *i* from outside the network, rather than through links or referrals from other nodes.

The second term, the endogenous (endo) component, captures the fact that past events increase the likelihood of future ones. Specifically, an event of type *j* at time $t_{k}^{j}<t$ contributes to the intensity of type *i* at time *t* through two factors. First, the average fertility coefficient $n_{i,j}$ denotes the expected number of type-*i* events directly triggered by a single event of type *j*, quantifying the average causal influence from *j* to *i*. As we will see below, the branching ratio matrix, defined through as $N=\{n_{i,j}{\}}_{i,j=1}^{M}$, recovers the adjacency matrix as a special limit case. Second, this influence decays over time according to a normalized kernel $\phi (u)=\frac{1}{\tau }e^{-u/\tau }\;1_{\left\{u>0\right\}}$, where $u=t-t_{k}^{j}$ denotes the elapsed time since the triggering event and τ>0 is a memory scale. The kernel is normalized according to $\int_{0}^{\infty }\phi (u)\;du=1$. While exponential decay is a common choice, other shapes such as power laws can be used to model systems with long memory. The scalar *branching ratio n*, defined as the spectral radius of the matrix *N*, must satisfy 0≤n<1 to ensure that the Hawkes process considered here is well-defined and nonexplosive. Unlike heuristic temporal-network centralities based on evolving adjacency matrices and prescribed decay rules, the Hawkes formulation derives temporal influence directly from a generative event process calibrated on observed event sequences.

Because the intensity $\lambda_{i}(t)$ represents the instantaneous expected rate of events of type *i*, it provides a natural operational measure of real-time activity, and hence of importance. A node with a higher $\lambda_{i}(t)$ is more likely to generate the next event and should therefore be ranked as more influential at that moment. For webpage ranking, $\lambda_{i}(t)$ represents the instantaneous rate at which page *i* is visited, providing a direct and interpretable measure of its current relevance. In social media applications, $\lambda_{i}(t)$ may represent the expected posting or resharing rate of an influencer, identifying who is currently driving engagement. In finance, $\lambda_{i}(t)$ corresponds to the instantaneous trading intensity of an asset, ranking instruments by their momentary contribution to market activity. In neuroscience, it can be interpreted as the firing rate of a neuron, highlighting those most actively shaping network-level dynamics. HawkesRank formalizes this interpretation by treating the SEMHP intensities $\lambda_{i}(t)$ themselves as dynamic rankings, yielding a time-resolved notion of influence that evolves with the system. Classical recursive centralities emerge only after temporal or ensemble averaging of these dynamics, whereas HawkesRank tracks the realized pathwise evolution of activity conditional on the observed history. Because these intensities can be estimated directly from empirical event data, HawkesRank provides an operational reference against which the performance of static centrality measures can be systematically evaluated.

### Katz centrality: a special case of HawkesRank

First, we show that Katz centrality and related classical measures arise naturally as stationary mean-field limits of HawkesRank. This correspondence serves both as a consistency result and as a conceptual bridge between classical recursive centralities and event-driven stochastic dynamics. Katz centrality is motivated by the idea that a node’s importance is determined recursively by the importance of its neighbors, augmented by an exogenous baseline contribution.

Formally, let $A\in R^{M\times M}$ be a nonnegative adjacency matrix, where $A_{ij}\geq 0$ quantifies the strength of the directed influence from node *j* to node *i*. Denote by $\lambda_{\max}$ the spectral radius of *A*, and let *α* satisfy $0<\alpha <1/\lambda_{\max}$ to ensure convergence. Given an exogenous weight vector $\beta \in R^{M}$, the Katz centrality vector $c^{\mathrm{katz}}$ is defined implicitly by


(2) $$c^{\mathrm{katz}}=\alpha Ac^{\mathrm{katz}}+\beta ,$$

with the closed-form solution


(3) $$c^{\mathrm{katz}}=(I-\alpha A{)}^{-1}\beta .$$

The vector *β* thus represents an exogenous source of importance, while the recursive term captures endogenous accumulation of influence through the network structure. Choosing $\beta =\vec{1}$ recovers the classical definition where all nodes contribute equally. In the limiting case $\alpha \to 1/\lambda_{\max}$, Katz centrality converges to eigenvector centrality. Finally, PageRank is a normalized variant of Katz centrality, obtained by applying the Katz formulation to the column-normalized version of *A* (see Methods for details).

We now calculate the first moment of the SEMHP with respect to all possible stochastic realizations of the process. Taking expectations on both sides of 1, we obtain


(4a ) $$E[\lambda_{i}](t)=E[\mu_{i}(t)]+\sum_{\;j=1}^{M}\int_{-\infty }^{t}ds\;E[\lambda_{j}](s)n_{i,j}\phi (t-s),$$

where $E[\lambda_{i}](t)$ refers to the ensemble average of the stochastic intensities $\lambda_{i}(t)$ across all realizations of the process (Methods). This ensemble-average quantity should be distinguished from the realized conditional intensity path $\lambda_{i}(t)$ associated with a particular observed event history. When the memory parameter *τ* approaches 0, the function ϕ(t) approaches a Dirac delta function, such that


(4b ) $$E[\lambda_{i}](t)=E[\mu_{i}(t)]+\sum_{\;j=1}^{M}E[\lambda_{j}](t)n_{i,j}.$$

Under these assumptions, HawkesRank subsumes Katz centrality in expectation: the expected HawkesRank rate obeys the Katz fixed-point relation under the following conditions. (i) the exogenous weight vector is time independent and nonstochastic, $E[\mu_{i}(t)]=\mu_{i}(t)\equiv \beta_{i}$; (ii) the branching ratio matrix $N=\{n_{i,j}{\}}_{\;j,i=1}^{M}$, where $n_{i,j}$ denotes the expected number of type-*i* events directly triggered by a single type-*j* event, is equal to the adjacency matrix αA.

**Figure 1:**
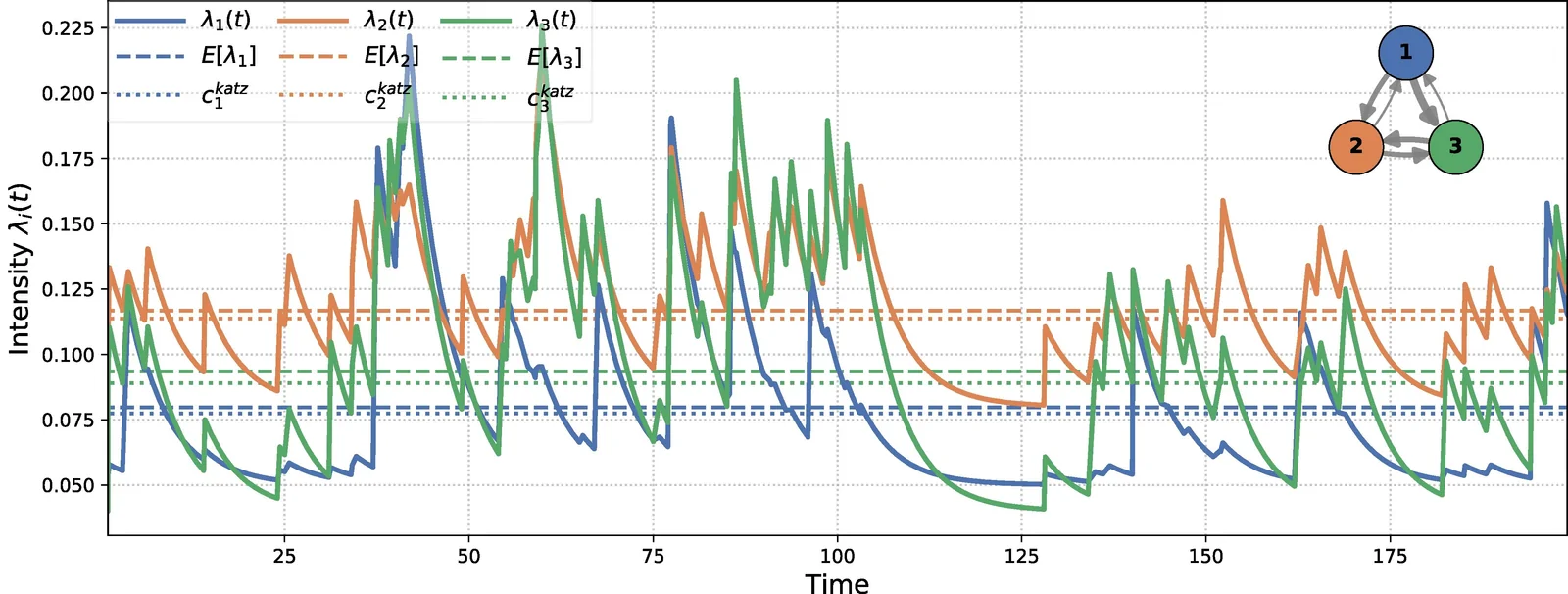
Temporal evolution of the event intensities $\lambda_{i}(t)$ for i=1,2,3 in a 3D Hawkes process. The branching ratio matrix *N* is illustrated in the top-right corner as a weighted network, where edge thickness indicates the strength of excitation between nodes. The intensities evolve dynamically in response to exogenous inputs with baseline values $\left(\mu_{1},\mu_{2},\mu_{3})=(0.05,0.08,0.04\right)$, generating endogenous bursts of activity through self- and cross-excitation. As a result, the relative ranking of the intensities changes over time, with the ordering of $\lambda_{1}$, $\lambda_{2}$, and $\lambda_{3}$ fluctuating throughout the simulation. By contrast, the first-moment Hawkes solution from Eq. 4a and the Katz centralities from Eq. 3 remain constant over time.

For static $\mu_{i}$, Katz centrality is also recovered as the long-term average of $\lambda_{i}(t)$ for arbitrary finite values of *τ* (Methods). Classical Katz centrality therefore corresponds to an averaged stationary description in which transient fluctuations, shocks, and path-dependent cascades are integrated out. HawkesRank thus generalizes Katz by allowing heterogeneous and explicitly time-dependent exogenous contributions $\beta_{i}(t)$. As we show below, it also provides a principled way to recover the interaction matrix *N* directly from event data, in contrast to traditional approaches where the adjacency matrix *A* is constructed heuristically and depends on modeling choices, such as the time scale over which interactions are aggregated. The resulting intensities $\lambda_{i}(t)$ define dynamic rankings that evolve through the interplay of exogenous input $\mu_{i}(t)$ and heterogeneous endogenous interactions encoded in *N*. This gives rise to punctuated bursts of activity, in contrast to the constant expectation $E[\lambda_{i}]$ and the static baseline implied by Katz centrality (Fig. 1). HawkesRank thereby embeds classical recursive centralities within a broader dynamic event-driven framework capable of representing temporally evolving influence and exogenous activity. Unlike temporal centralities constructed through prescribed decay rules on evolving adjacency matrices, the Hawkes formulation derives these dynamics from a generative stochastic process calibrated on observed event sequences.

### The importance of dynamic rankings

To illustrate the practical implications of event-driven rankings, we generate data from the SEMHP (Eq. 1) for M=10 types of events. The exogenous intensities are drawn from a power law, $\mu_{i}=i^{-1/2}$, such that nodes with lower indices exhibit systematically higher baseline activity.

The branching ratio matrix *N* is constructed in three steps. First, we sample an unweighted adjacency matrix from the Barabási–Albert ensemble, capturing the mechanism of preferential attachment commonly observed in real-world systems. Combined with the Hawkes event dynamics, this construction provides a generic model of interacting systems with endogenous cascades, heterogeneous activity, and temporally evolving influence. Nodes are added sequentially in the order i=1,…,M, and each newly added node establishes η=5 outgoing edges, attaching to existing nodes with probability proportional to their current degree. Self-loops are included so that diagonal elements $n_{ii}>0$ represent genuine auto-reinforcement, a defining feature of self-exciting processes. Second, weights are assigned to the nonzero entries of *N* by independently sampling them from the distribution on [0,1]. Third, the matrix is rescaled to achieve a target branching ratio of n=0.6 by multiplying each nonzero weight by $n/\lambda_{\max}$.

We fix the memory kernel decay parameter at τ=1, so that the effective time scale of fluctuations is ≈1/(1−n).

Given these parameters, we compute the intensities $\lambda_{i}(t)$ on a fine-grained time grid over T=200 time steps. This yields a sequence of instantaneous reference rankings induced by the realized conditional intensities $\lambda_{i}(t)$ that reflect the system’s evolving activity: At time *t*, event type *i* is ranked above event type *j* whenever $\lambda_{i}(t)>\lambda_{j}(t)$. We then assess how well four static centrality benchmarks approximate these dynamic rankings: (i) First-moment Hawkes ranking (Eq. 4b) using the true $\mu_{i}$; (ii) Katz centrality (Eq. 2) with $\beta =\vec{1}$ instead of proper $\mu_{i}$; (iii) Eigenvector centrality, obtained as the limiting case of Katz as $\alpha \to 1/\lambda_{\max}$; (iv) PageRank, which modifies (ii) by replacing the adjacency matrix A=N with its column-normalized counterpart. As an additional sensitivity analysis, the Supplementary material further compares HawkesRank against temporal-network centrality measures (29, 30).

**Figure 2:**
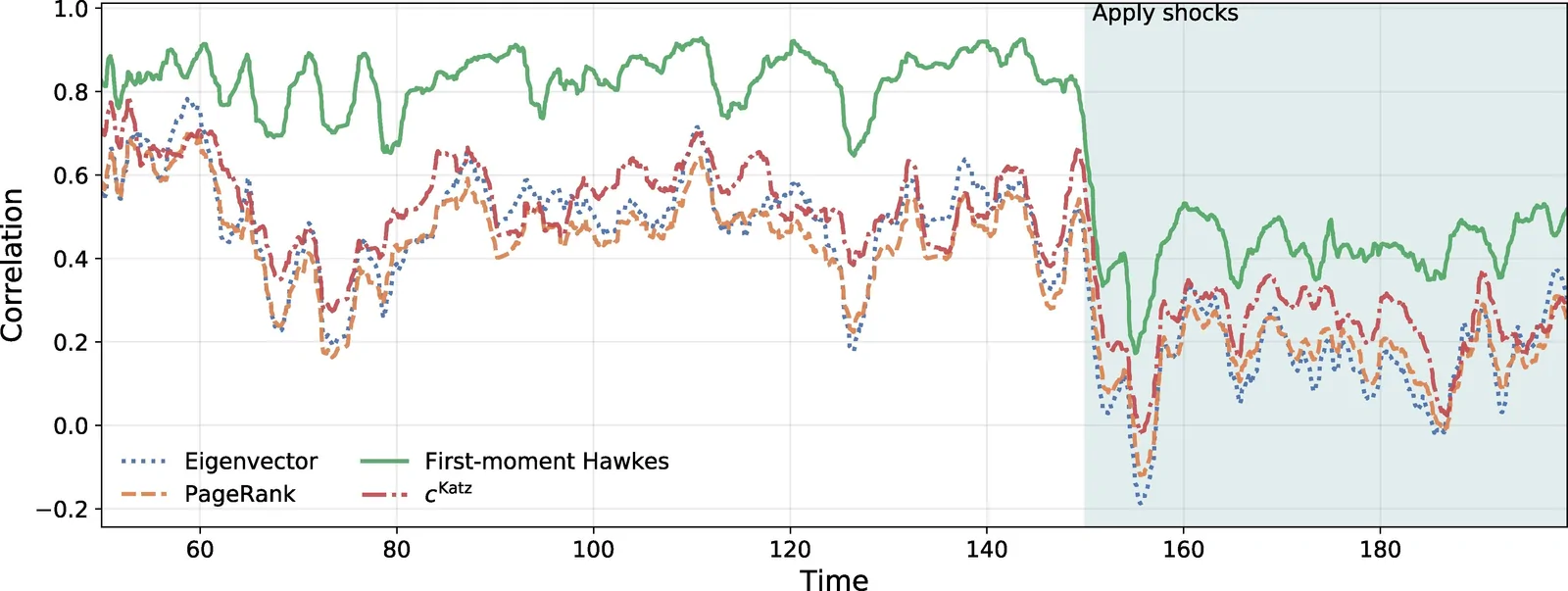
For each of four static centrality measures, we compute the Spearman rank correlation between the corresponding static ranking and the ground-truth ranking induced by the instantaneous intensities $\{\lambda_{i}(t){\}}_{i=1}^{M}$ at each time *t*. Higher correlation values indicate better agreement between the static and dynamic rankings. The vertical dashed line at t=150 marks the onset of an exogenous shock, implemented by increasing the smallest baseline intensity $\mu_{10}$ by a factor of 10. The figure shows one representative realization of the SEMHP simulation. The correlation curves are smoothed using a centered moving average over 30 consecutive points of the intensity evaluation grid to reduce high-frequency stochastic fluctuations in the event-driven rankings. Ensemble robustness over 100 independent realizations is reported in the Supplementary material.

For each benchmark, we compute the Spearman rank correlation between its static ranking and the ground-truth ranking at each time step *t*. This yields a time series of correlation coefficients for each centrality measure, quantifying their correspondence with the evolving intensity dynamics of the system (Fig. 2). Among the static approximations considered here, the first-moment Hawkes ranking exhibits the highest average correspondence, followed by conventional Katz centrality with homogeneous exogenous terms. This underscores the importance of accounting for exogenous influence, particularly its heterogeneity. In other words, the objective of this experiment is not to establish an absolute notion of ranking performance, but rather to quantify how much information about temporally evolving activity is lost when dynamic event-driven systems are approximated by stationary centrality measures.

The results are robust to structural parameter choices. Qualitatively similar patterns are observed when varying *η* from 1 to 8 and the branching ratio *n* from 0.3 to 0.9. These findings expose a fundamental limitation of static ranking measures. Even the best-performing method (the first-moment Hawkes approximation) displays time-varying fluctuations in rank correlation, indicating that static approaches are insufficient to capture the dynamics of evolving systems. Importantly, this limitation persists even for the first-moment Hawkes approximation, highlighting the distinction between stationary ensemble averages and realized pathwise dynamics.

The need for dynamic importance rankings becomes even more evident when exogenous drivers fluctuate in response to shocks or structural changes. In social media systems, exogenous attention may shift abruptly following breaking news, viral content, or platform-level interventions, temporarily elevating the activity of specific users or topics. In financial markets, external information such as earnings announcements, macroeconomic releases, or regulatory actions can induce sudden changes in trading intensity across assets. In neuroscience, external stimuli or experimental perturbations can transiently modulate neuronal firing rates, altering the relative importance of neurons over time.

To evaluate ranking performance under such conditions, we introduce an exogenous shock to the baseline intensity *μ*. Specifically, at time t=150, the smallest exogenous intensity, $\mu_{10}$, is increased 10-fold and maintained at this elevated level for 50 time steps. This intervention alters the exogenous activity landscape and thereby changes the evolving hierarchy of conditional intensities. Figure 2 shows that once the shock occurs, the correlations of all static benchmarks exhibit higher variability and a pronounced decline in agreement with the true rankings. The first-moment Hawkes ranking provides only limited improvement over the other static methods. Although it incorporates exogenous contributions, it assumes them to be time-invariant and therefore cannot capture the time-varying external drivers that characterize real-world systems. This highlights a key limitation of static centrality measures: they cannot adapt to shocks or represent transient dynamics, resulting in less reliable rankings when the system undergoes perturbations.

Since the reference ranking is defined by the realized conditional intensities of the underlying event-driven process, Fig. 2 quantifies how much ranking information is lost when temporally evolving dynamics are approximated by static or stationary descriptions.

### Dynamically ranked emotions

Emotional contagion in online communication provides a natural setting for evaluating dynamic ranking methods. Messages arrive as discrete events, emotional responses arise from both external stimuli and interactions among participants, and the prominence of emotions evolves rapidly over time. This makes emotional communication systems particularly challenging for static ranking approaches that assume time-invariant influence structures. To showcase the benefits of HawkesRank, we thus analyze emotional dynamics in YouTube live-chat discussions. YouTube Live allows viewers to post messages in real time while watching a streaming video, producing a continuous stream of time-stamped chat messages that reflect audience reactions to the unfolding content.

Following the approach detailed in Ref. (43), emotions expressed in chat messages are categorized according to the widely used six basic emotion framework consisting of joy, surprise, anger, disgust, fear, and sadness (45). Each message is represented as a 6D binary vector indicating the nonexclusive presence of these emotions. Our objective is to rank the prevalence and influence of these emotions over time, identifying which emotions dominate the conversation at each moment.

**Figure 3:**
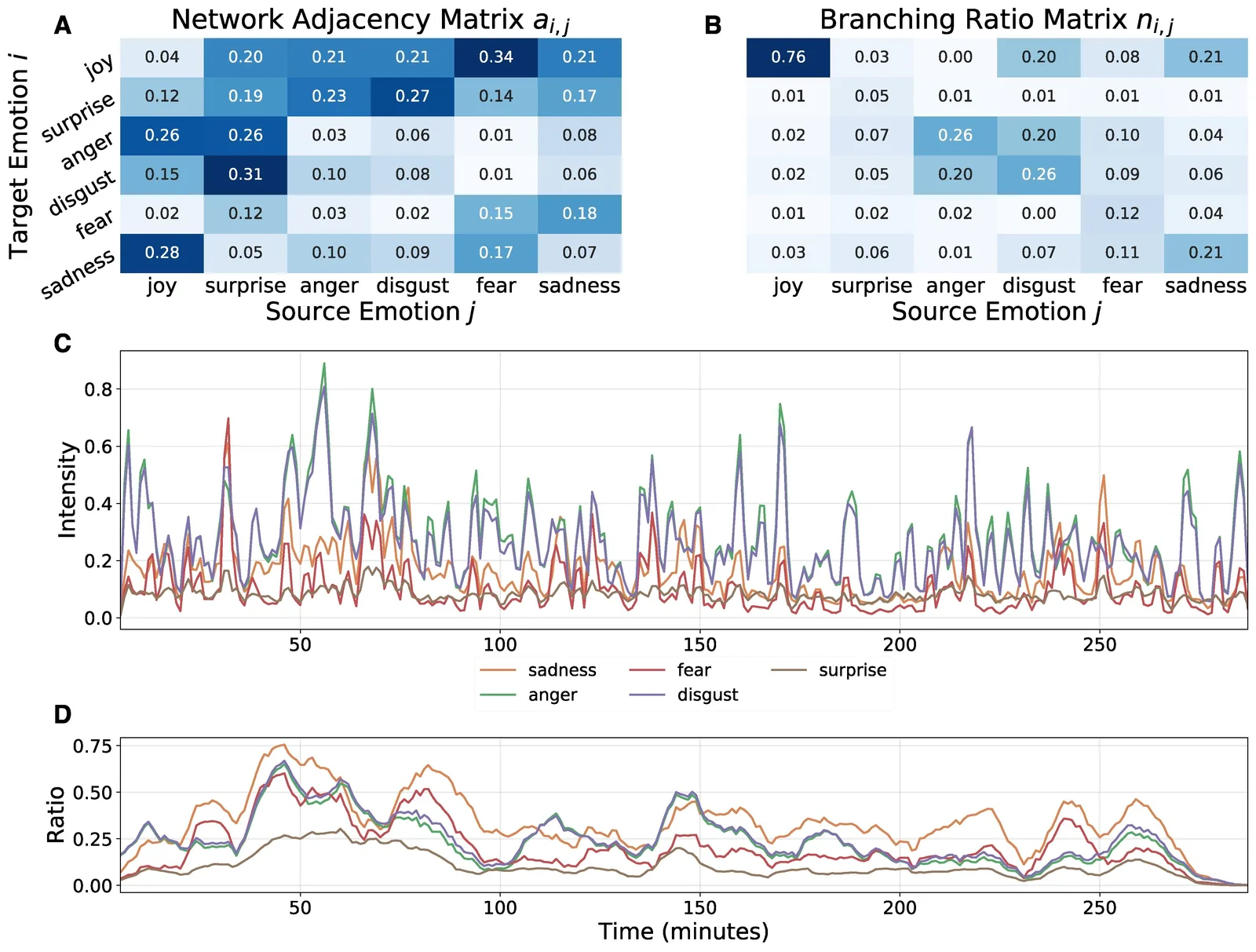
Emotion dynamics in a YouTube live-chat video. A) Network adjacency matrix, where entry (i,j) quantifies the influence of source emotion *j* on target emotion *i*. B) Estimated branching ratio matrix *N*, where entry (i,j) quantifies the influence of source emotion *j* on target emotion *i*. C) Hawkes intensity trajectories $\lambda^{i}(t)$ for five basic emotions across the video. The sixth emotion (*joy*) dominates across emotions and is therefore omitted from plots for visual clarity. The intensities are smoothed using a centered two-time-step moving average. D) Time-varying ratio of endogenous activity, defined as the proportion of self- and cross-excitation relative to total intensity. The ratios are smoothed using a centered 10-time-step moving average.

A common approach to analyzing emotional influence is to construct a directed network from message activity using lead–lag correlations between emotion time series. Chat messages are first aggregated into temporal bins of *b* minutes, yielding time series $x_{i}(t)$ that record the number of occurrences of emotion *i* in bin *t*. For each pair of emotions (i,j), we compute the Pearson correlation between $x_{j}(t)$ and $x_{i}(t+\ell b)$, where ℓ is an integer lag indicating how many bins separate the two observations. The adjacency entry $A_{i,j}$ is defined as this lagged correlation, so that an edge j→i indicates that fluctuations in emotion *j* tend to precede fluctuations in emotion *i* after ℓ bins. Aggregating these relations and normalizing the resulting matrix by its Frobenius norm yields the adjacency matrix shown in Fig. 3A.

This network construction introduces two free parameters, the bin width *b* and the lag ℓ, whose choice is not determined by the data. In Fig. 3, we have set b=0.5 and ℓ=2. However, the inferred network structure $A_{ij}$ can vary substantially across plausible parameter values (Supplementary material). In contrast, the HawkesRank framework estimates excitation effects directly from the event timestamps, inferring the interaction structure statistically from event timestamps without requiring heuristic aggregation windows or lag-selection rules to first construct a static interaction network. We therefore model the emotional dynamics using a multivariate Hawkes process. In this framework, the occurrence of an emotion in a chat message is treated as a discrete event, and emotional propagation is captured through self- and cross-excitation mechanisms. Each emotion *i* is characterized by an intensity $\lambda_{i}(t)$ representing the instantaneous rate at which that emotion appears in the chat stream. According to Eq. 1, the branching ratio matrix *N* captures endogenous excitation whereby past emotional expressions influence future ones. All parameters are fitted using maximum log-likelihood. Additional modeling and estimation details are provided in Ref. (43).

The estimated branching matrix *N*, shown in Fig. 3B, differs markedly from the adjacency matrix derived from the static network construction, even though both are based on the same underlying data. In particular, the Hawkes estimation reveals strong self-excitation effects, consistent with prior evidence of emotional contagion in online interactions (46). It also identifies a pronounced bidirectional interaction between anger and disgust that is not captured by the adjacency-based network. Conversely, the static network construction assigns joy as a dominant source of all other emotions, an interpretation that is behaviorally implausible since expressions of joy rarely induce fear or surprise. The Hawkes framework disentangles these relationships by separating endogenous excitation from exogenous drivers and by estimating the interaction structure directly from the event data.

As an additional validation, we also evaluated the predictive performance of the inferred interaction structure on held-out YouTube Live data (Supplementary material). Although HawkesRank is primarily intended as a dynamic ranking framework rather than a predictive model, it substantially outperforms the corresponding lead–lag correlation-network representation in predicting the next observed emotion. The geometric mean probability assigned to the emotion that actually occurs is ≈2.3 times higher under HawkesRank, providing independent out-of-sample support for the inferred interaction structure.

Next, we analyze the evolution of the HawkesRank rankings $\lambda_{i}(t)$ for the six emotions (Fig. 3C). The intensity of the emotion *joy* dominates throughout the observation period and is therefore omitted from the plot to preserve visual resolution. The remaining five emotions exhibit substantial temporal fluctuations, abrupt changes, and frequent crossings between trajectories. These dynamics demonstrate that the relative prominence of emotions changes continuously during the video, highlighting the limitations of static ranking approaches.

Finally, HawkesRank enables a decomposition of total activity into endogenous and exogenous contributions. Emotions in YouTube Live chats are particularly suitable for such a decomposition because the video itself provides a strongly time-varying exogenous driver. For example, dramatic or humorous moments in the video may trigger bursts of specific emotional reactions in the chat. The HawkesRank framework therefore provides a statistical decomposition between activity attributed to the modeled exogenous component and activity attributed to endogenous amplification among viewers. Figure 3D shows that the endogenous share of emotional activity varies considerably over time, indicating that the importance of internal amplification relative to external stimulation is itself dynamic. Such a decomposition is not available within the static network formulation, yet it provides additional insight into the mechanisms underlying emotional dynamics. More broadly, the framework enables alternative ranking schemes based on total activity, purely exogenous influence, or endogenous amplification.

Together, these findings demonstrate that static centrality measures are insufficient for characterizing and evaluating inherently dynamic systems. By collapsing interactions over time, static network approaches obscure temporal heterogeneity and evolving influence patterns. In contrast, the HawkesRank framework explicitly models time-dependent excitation dynamics, yielding instantaneous and adaptive rankings that update dynamically in response to realized events and evolving interaction patterns.

More generally, the empirical analysis illustrates the distinction between static structural centralities and rankings based on conditional event dynamics. The latter retain information about transient amplification, abrupt shocks, and temporally localized influence patterns that are averaged out in stationary network representations.

## Methods

### Classical centrality measures

To benchmark our approach, we consider three widely used centrality measures in network science: eigenvector centrality, Katz centrality, and PageRank.

#### Eigenvector centrality

Eigenvector centrality is based on the principle that a node is important if it is connected to other important nodes. For a network with adjacency matrix $A_{ij}$, the centrality score $c_{i}$ satisfies


(5) $$c_{i}^{\left(\mathrm{eig}\right)}=\frac{1}{\lambda }\sum_{\;j=1}^{M}A_{ij}c_{j}^{\left(\mathrm{eig}\right)},$$

which in vector form corresponds to the eigenvalue problem


(6) Ac=λc,

where *λ* is the largest eigenvalue of *A*.

#### Katz centrality

Katz centrality extends eigenvector centrality by incorporating both direct and indirect connections. Let $(A^{k}{)}_{ij}$ denote the number of walks of length *k* from node *j* to node *i*, and let *α* be a damping factor that discounts longer paths. The Katz centrality score is defined as


(7) $$c_{i}^{\left(\mathrm{katz}\right)}=\sum_{k=1}^{\infty }\sum_{\;j=1}^{M}\alpha^{k}(A^{k}{)}_{\;ij}.$$

Equivalently, it can be written in recursive form as


(8) $$c_{i}^{\left(\mathrm{katz}\right)}=\alpha \sum_{\;j=1}^{M}A_{ij}c_{j}^{\left(\mathrm{katz}\right)}+\beta_{i},$$

where $\beta_{i}$ represents exogenous contributions to node importance. Convergence requires α<1/λ, where *λ* is the largest eigenvalue of *A*.

#### PageRank

PageRank is a variant of eigenvector centrality designed for directed networks such as the web (3). The PageRank score of node *i* is defined as


(9) $$\mathrm{PR}(i)=(1-d)\frac{1}{M}+d\sum_{\;j\in M(i)}\frac{1}{L(j)}\mathrm{PR}(j),$$

where M(i) is the set of nodes linking to *i*, L(j) is the number of outgoing links from node *j*, and *d* is a damping factor typically set to 0.85. The first term represents random jumps across the network, ensuring that every node has a nonzero probability of being visited.

Additional discussion and mathematical details are provided in the Supplementary material.

### Multivariate Hawkes process

Consider *M* event types labeled i=1,…,M, where each type corresponds to events occurring on a particular object (e.g. a webpage, user, or asset). The occurrence of events is characterized by an intensity function $\lambda_{i}(t)$, which specifies the instantaneous rate at which events of type *i* occur at time *t*. The probability of observing an event of type *i* in a short interval [t,t+dt) is $\lambda_{i}(t)\;dt$. The multivariate Hawkes process is defined as


(10) $$\lambda_{i}(t)=\mu_{i}(t)+\sum_{\;j=1}^{M}\int_{-\infty }^{t}f_{i,j}\phi_{i,j}(t-s)\;dN_{j}(s),$$

where $N_{j}(s)$ denotes the counting process of events of type *j* up to time *s*.

The first term $\mu_{i}(t)$ represents the exogenous or background rate of events, capturing activity driven by external factors or intrinsic attractiveness. The second term describes endogenous excitation, whereby past events increase the likelihood of future events.

Two elements determine the strength and timing of this excitation. First, the coefficient $f_{i,j}$ represents the number of type-*i* events triggered by a single event of type *j*, capturing the strength of cross-excitation between event types. Second, the kernel $\phi_{i,j}(t)$ describes how the influence of past events decays over time. In our implementation, we use an exponential kernel $\phi_{i,j}(t)=\exp\;(-t/\tau_{i,j})/\tau_{i,j},$ which defines a characteristic memory timescale $\tau_{i,j}$.

The expected number of first-generation events of type *i* generated by an event of type *j* is given by $n_{i,j}=E[f_{i,j}],$ and the collection of these coefficients forms the branching ratio matrix $N=(n_{i,j}).$ The spectral radius of *N*, denoted *n*, determines the stability of the process (47).

For analytical tractability, we consider the commonly used mean-field formulation in which fertilities are fixed at their expectations, $f_{i,j}=n_{i,j}$. Under this assumption, the intensity reduces to


(11) $$\lambda_{i}(t)=\mu_{i}(t)+\sum_{\;j=1}^{M}\int_{-\infty }^{t}n_{i,j}\phi (t-s)\;dN_{j}(s),$$

which corresponds to the formulation used in the Results section.

### First-moment representation and connection to classical centrality

The Hawkes process formulation admits a direct connection to classical network centrality measures through its first moment. Taking the expectation of the intensity in Eq. 11 over all stochastic realizations yields the mean-field equation


(12) $$E[\lambda_{i}](t)=\mu_{i}(t)+\sum_{\;j=1}^{M}\int_{-\infty }^{t}E[\lambda_{j}](s)n_{i,j}\phi (t-s)\;ds.$$

This equation describes how the expected activity of each entity results from both exogenous inputs and cascades generated through the interaction matrix $N=(n_{i,j})$.

Two limiting regimes connect this formulation to classical centrality metrics. First, when the memory kernel becomes infinitely short (τ→0), the kernel approaches a Dirac delta function and Eq. 12 reduces to


(13) $$E[\lambda_{i}](t)=\mu_{i}(t)+\sum_{\;j=1}^{M}n_{i,j}E[\lambda_{j}](t).$$

In vector form this yields the solution


(14) $$E[\Lambda ]=(I-N{)}^{-1}\mu .$$

Equation 14 is equivalent to the recursive form


(15) E[Λ]=μ+NE[Λ],

which directly parallels the Katz formulation from Eq. 8. Identifying $\mu =\vec{\beta }$ and N=αA therefore shows that the stationary first moment of the Hawkes process recovers Katz centrality.

The same stationary expression also arises in the long-time limit of the Hawkes process when the exogenous intensity μ(t) is constant. In that case, the convolution term in Eq. 12 converges to a steady value and the expectation becomes time-independent, yielding the fixed-point solution Eq. 14. Full derivations are provided in the Supplementary material.

## Discussion and conclusions

We introduced HawkesRank, a dynamic framework for measuring importance in evolving systems based on multivariate Hawkes point processes. In this formulation, importance is interpreted as the instantaneous event intensity $\lambda_{i}(t)$, i.e. the expected rate at which events associated with an entity occur. This gives ranking a direct interpretation in terms of observable system activity and links centrality to event-generation dynamics. Within this framework, widely used measures such as Katz centrality, eigenvector centrality, and PageRank emerge as static mean-field limits, placing classical centrality metrics within a broader event-driven model of influence. This interpretation clarifies the assumptions under which stationary recursive centralities emerge as effective descriptions of temporally evolving interaction systems.

Beyond this unification, HawkesRank offers several conceptual advantages. It separates exogenous drivers, representing activity not explained by endogenous network excitation within the specified model, from endogenous amplification generated through interactions. At the same time, the branching ratio matrix *N* provides a statistically inferred interaction structure, providing an alternative to heuristic network constructions by estimating interaction patterns directly from event data. Together, these features transform ranking from a purely structural property of networks into a dynamic, interpretable model of event generation that naturally adapts to shocks, feedback, and temporal evolution.

It is important to stress that, under the standard assumptions of parametric Hawkes-process models, the HawkesRank decomposition can be statistically inferred from a single sufficiently long multivariate realization. The unobserved genealogy of the process can be viewed as a latent structure: each event is associated with a probability of being exogenous and probabilities of being triggered by each compatible past event. Aggregating these attribution probabilities yields estimates of the expected number of first-generation offspring from type *j* to type *i*, and therefore of the branching matrix *N*. Practical identifiability requires nonexplosivity, ρ(N)<1, sufficient event counts, sufficiently strong excitation, and a specified or regularized form of the exogenous intensity $\mu_{i}(t)$. If $\mu_{i}(t)$ is allowed to vary arbitrarily, the separation between exogenous bursts and endogenous cascades becomes ill-posed; conversely, if $\mu_{i}(t)$ is too rigid, exogenous nonstationarity may be misattributed to endogenous excitation. The HawkesRank estimates should therefore be interpreted conditional on the chosen model class for $\mu_{i}(t)$, the kernel family, and the observation window.

Concerning sensitivity to the specification of the exogenous component, the HawkesRank endo–exo decomposition should not be interpreted as a sequential residual construction in which the endogenous component is fitted first and the exogenous component is whatever remains unexplained. Instead, $\mu_{i}(t)$ and *N* are estimated jointly from the full event history. In latent-branching formulations of Hawkes estimation, each event can be probabilistically attributed either to the exogenous background or to endogenous triggering by previous events, while direct likelihood-based estimation jointly calibrates the same model components. This joint estimation is central to the ability of Hawkes models to address the endo–exo attribution problem (48, 49). Nevertheless, the specification of $\mu_{i}(t)$ remains important. If $\mu_{i}(t)$ is constrained to be too rigid, for example constant when the true background is strongly time-varying, exogenous bursts may be falsely attributed to endogenous amplification, leading to overestimation of *N* and apparent near-criticality. Conversely, flexible adaptive parametrizations of $\mu_{i}(t)$, such as adaptive log-spline models, have been shown in synthetic tests to recover complicated exogenous structures with good stability and to improve endo–exo attribution (48, 49). We therefore view the flexibility of $\mu_{i}(t)$ not as a weakness of HawkesRank but as an essential modeling ingredient: exogenous activity and endogenous amplification must be estimated together.

Several directions remain for future work. On the modeling side, richer excitation structures could be explored by moving beyond simple exponential kernels toward nonparametric or multiscale memory functions, while also allowing for inhibition, competition, and time-varying interaction matrices N(t). From a computational perspective, scalable online algorithms with uncertainty quantification will be essential for applying HawkesRank to large systems containing millions of entities. This challenge is particularly relevant for current likelihood-maximization-based calibration methods, whose computational cost can become prohibitive as the number of interacting dimensions increases. However, this limitation also opens the door to more scalable alternatives, such as stochastic-gradient estimation, online filtering, moment-based inference, variational approximations, or neural/low-rank parametrizations of the excitation kernel. In large-scale applications, these approaches should be tested in combination with sparse, regularized, or low-rank structures to exploit the fact that most entities interact only with a small subset of the system. Theoretical advances are likewise needed to establish identifiability conditions, sample-complexity bounds, and robustness guarantees for estimating $\mu_{i}(t)$, N(t), and the associated rankings from event data.

Expanding empirical applications will further clarify the practical value of the framework. In financial markets, HawkesRank may provide a framework for studying periods in which endogenous amplification dominates baseline activity. In social media, it may help characterize the relative contribution of endogenous amplification and externally driven visibility. In science, it may help study the relationship between cumulative amplification dynamics and baseline citation activity. In public health, it may help disentangle persistent transmission dynamics from transient bursts associated with super-spreading events.

By grounding ranking in event intensities and explicitly modeling memory and feedback, HawkesRank provides an interpretable, data-driven, and adaptive notion of importance. We view this event-based perspective as a foundation for the next generation of ranking systems, capable of modeling dynamically evolving influence in complex systems.

## Ethics approval and data collection

This study was approved by the Science and Technology Ethics Committee of the Southern University of Science and Technology (reference number 20250407).

The YouTube Live chat data analyzed here, collected as described in Ref. (43), consist exclusively of messages that are publicly viewable by any visitor to the corresponding live stream, without requiring login, registration, or any other form of privileged access. Because data collection involved no direct interaction with, or recruitment of, the individuals who posted these messages, and because the messages were already public at the time of collection, informed consent was not required. Our analysis is conducted at the level of message-level emotion categories and aggregate statistics; no usernames or other personally identifying information are used or reported.

## Supplementary Material

pgag292_Supplementary_Data

## Data Availability

Publicly available data were obtained from YouTube. The data and code required to reproduce the analyses are available at: https://github.com/ivylyis/Quantification-of-the-Self-Excited-Emotion-Dynamics-in-Online-Interactions.

## Preprints

This manuscript was posted as a preprint at https://arxiv.org/html/2603.11472v1.
