# Supplementary material for: HawkesRank: Event-driven centrality for real-time importance ranking
Source: PNAS Nexus. 2026 Sep 1;5(9):pgag292. doi: 10.1093/pnasnexus/pgag292 (PMC13560081; doi:10.1093/pnasnexus/pgag292)
Supplement: Supplementary file 1 [file pgag292_supplementary_data.pdf]

Supplementary Material for:

HawkesRank: Event-Driven Centrality for Real-Time Importance Ranking

This Supplementary Material accompanies the main text and provides: a review of classical centrality measures and their limitations (Section S1); the derivation showing that the stationary first moment of the self-exciting multivariate Hawkes process (SEMHP) recovers Katz centrality (Section S2); a sensitivity comparison against temporal-network ranking methods (Section S3); an ensemble robustness check of the simulation study (Section S4); an analysis of the sensitivity of correlation-based emotion networks to construction parameters (Section S5); and an out-of-sample predictive validation of HawkesRank on the YouTube Live chat data (Section S6).

S1 Review of Centrality Measures

This appendix reviews the standard definitions and limitations of three widely used centrality metrics: eigenvector centrality, Katz centrality, and PageRank, all of which are relevant to our work and closely related to one another.

S1.1 Definitions of Centrality Measures

We begin by defining each centrality measure in its classical form, highlighting key mathematical properties and connections.

Eigenvector Centrality. Eigenvector centrality is based on the self-consistent principle that a node is important if it is connected to other important nodes. For a network with adjacency matrix A_{ij}, the eigenvector centrality of node i is given by

$$c_i^{(\text{eig})} = \frac{1}{\lambda} \sum_{j=1}^{N} A_{ij} c_j^{(\text{eig})}, \tag{S1}$$

where λ is the largest eigenvalue of the matrix A. In vector form, this corresponds to the standard eigenvector equation:

$$Ac = \lambda c. \tag{S2}$$

The score c_i is thus proportional to the sum of the centralities of its neighbors. This centrality is computationally efficient and forms the foundation for more refined variants, including Katz centrality and the PageRank algorithm.

Katz Centrality. Katz centrality generalizes eigenvector centrality by accounting not only for immediate neighbors but also for indirect connections of all lengths. Let $(A^k)_{ij}$ denote the number of walks of length k from node j to node i, and let $\alpha \in (0, 1)$ be a damping factor that attenuates longer paths. Then the Katz centrality of node i is defined as:

$$c_i^{(\text{katz})} = \sum_{k=1}^{\infty} \sum_{j=1}^{N} \alpha^k (A^k)_{ij}. \tag{S3}$$

This represents a discounted sum over all walks leading to node i, assigning greater weight to shorter paths. Nodes that are easier to reach via many short paths receive higher centrality scores.

Defining the identity matrix of size N as I, and the all-ones vector as $\vec{I}$, this expression can be rewritten compactly as:

$$c^{\text{katz}} = \left(\sum_{k=1}^{\infty} (\alpha A)^k \right) \vec{I}. \tag{S4}$$

Using the geometric series identity, this becomes:

$$c^{\text{katz}} = \left((I - \alpha A)^{-1} - I \right) \vec{I}. \tag{S5}$$

Multiplying both sides by $(I - \alpha A)$ yields:

$$c^{\text{katz}}(I - \alpha A) = \vec{I} - (I - \alpha A)\vec{I} \tag{S6a}$$

$$= \alpha A \vec{I}. \tag{S6b}$$

Equivalently,

$$c^{\text{katz}} = \alpha A \vec{I} + \alpha A c^{\text{katz}} \tag{S7a}$$

$$= \alpha A (c^{\text{katz}} + \vec{I}), \tag{S7b}$$

which can be written component-wise as:

$$c_i^{(\text{katz})} = \alpha \sum_{j=1}^{N} A_{ij} (c_j^{(\text{katz})} + 1). \tag{S8}$$

To ensure that the matrix $(I - \alpha A)$ is invertible, we require $\alpha < 1/\lambda$, where λ is the largest eigenvalue of A. This condition ensures sufficient discounting of long paths to guarantee convergence.

The definition in Equations (S3) and (S4) assumes equal influence from all source nodes by using the uniform vector $\vec{I}$. A natural generalization replaces $\vec{I}$ with a vector $\vec{\beta}$, which allows for heterogeneous exogenous contributions:

$$c_i^{(\text{katz})} = \alpha \sum_{j=1}^{N} A_{ij} c_j^{(\text{katz})} + \beta_i. \tag{S9}$$

As $\vec{\beta} \to 0$ and $\alpha \to 1/\lambda$, Katz centrality converges to eigenvector centrality.

PageRank. The PageRank algorithm, originally developed to rank webpages, is a variant of eigenvector centrality adapted to directed hyperlink networks [1]. It assigns higher scores to nodes that are linked by other high-ranking nodes, while incorporating a damping factor to prevent rank sinks.

The PageRank of node i is defined by the recursive equation:

$$PR(i) = (1 - d)\frac{1}{N} + d \sum_{j \in M(i)} \frac{1}{L(j)} PR(j), \tag{S10}$$

where $M(i)$ is the set of nodes linking to i, and $L(j)$ is the number of outbound links from node j. The damping factor $d \in (0, 1)$, typically set to 0.85, represents the probability that a user follows a link rather than jumping to a random page.

The first term ensures that each page has a nonzero probability of being visited, mitigating the effects of disconnected or cyclic subgraphs. When $d = 1$, the formulation reduces to pure eigenvector centrality. PageRank has been widely adopted in domains beyond web search, including citation networks, social systems, protein interaction networks, and sports rankings [2].

S1.2 Limitations of Centrality Measures in Network Analysis

Classical centrality measures exhibit several critical limitations that constrain their applicability to real-world, dynamic systems. These include divergence from fundamental values, static formulations that ignore temporal evolution, and reliance on arbitrary or ad hoc parameters. Below, we synthesize these issues and review related extensions and partial remedies.

First, centrality-based rankings can deviate significantly from intrinsic value. Because these methods amplify network-based visibility, they are susceptible to manipulation and self-reinforcing distortions. Examples include search engine optimisation (SEO) and link farming, the artificial boosting of social media influencers, and the formation of echo chambers where visibility compounds independently of quality. Like financial markets, centrality systems can exhibit bubbles-phases where perceived importance diverges from fundamental worth, fueled by endogenous feedback rather than intrinsic merit. Valuable but less popular nodes are often eclipsed by highly connected but lower-quality content, reflecting an emphasis on influence over substance.

Second, most classical metrics are static. They capture the state of a network at a single moment and fail to account for ongoing changes in structure or context. In reality, node importance is highly dynamic: news outlets gain prominence during major events, influencers rise and fall with viral content, and geopolitical or market shifts suddenly reconfigure the strategic relevance of institutions or infrastructure. Static metrics overlook such fluctuations and risk producing rankings that are outdated, misleading, or blind to emerging threats and opportunities. Dynamic frameworks, such as the endo-exo ranking approach proposed here, address this by modeling both exogenous shocks and endogenous reinforcement over time.

Third, widely used metrics depend on arbitrary damping factors or weights. For instance, PageRank typically uses a damping factor of 0.85, chosen heuristically rather than derived from first principles [3]. More broadly, external information is often incorporated through uniform or ad hoc weight vectors, offering little interpretability or adaptability. Adjusting these parameters to fit new contexts requires costly manual tuning and trial-and-error. Moreover, static weights cannot respond to shifting dynamics or new data without re-specification.

Several extensions have been proposed to address these shortcomings. Time-aware adaptations such as Temporal PageRank [4, 5] and snapshot-aggregation methods [6, 7] introduce some temporal responsiveness but remain fundamentally based on static formulations. Continuous-time ODE models [8] generalize Katz centrality while avoiding discretization but lack stochastic dynamics, event-level resolution, or an explicit separation between endogenous and exogenous effects.

Recent data-driven models attempt to learn dynamic influence scores directly from observed cascades or metadata using machine learning or statistical inference [9–11]. While flexible and context-aware, such methods often require large annotated datasets, are computationally intensive, and lack interpretability. Moreover, they typically optimize for predictive performance rather than theoretical consistency or explanatory clarity.

Taken together, these observations point to a fundamental limitation of existing centrality metrics: they rely heavily on structural reinforcement while neglecting exogenous drivers and intrinsic value. As a result, they risk misrepresenting influence, misallocating attention and resources, and reinforcing systemic vulnerabilities. A principled alternative requires a shift toward dynamic, event-driven, and interpretable ranking systems that jointly model memory, network reinforcement, and exogenous signals. These goals are addressed by the HawkesRank framework developed in this work.

S2 Stationary First Moment of the SEMHP Recovers Katz Centrality

In this appendix, we present the theoretical result that the stationary first moment of the Hawkes process recovers Katz centrality (Eq. (S9)) as a special case. This mapping underscores the Hawkes process as a unifying framework for ranking in complex systems, while at the same time revealing the inherent limitations of traditional centrality measures.

S2.1 General Expression of the Multivariate Hawkes Process

We start by presenting the general form of the multivariate Hawkes process from which the simplified version used in the main text is derived. For reference, the simplified form of the intensity, introduced as Eq. (1) of the main text, is restated here as

$$\lambda_i(t) = \underbrace{\mu_i(t)}_{\text{exo}} + \underbrace{\sum_{j=1}^{M} \sum_{k:t_k^j<t} n_{i,j}\phi(t - t_k^j)}_{\text{endo}} \tag{S11}$$

First, let us note that the standard mathematical formulation in the language of point process replaces expression (S11) by

$$\lambda_i(t) = \underbrace{\mu_i(t)}_{\text{exo}} + \underbrace{\sum_{j=1}^{M} \int_{-\infty}^{t} \mathrm{d}N_j(s)\; n_{i,j}\phi\,(t - s)}_{\text{endo}}\,. \tag{S12}$$

In this formula, the variable $N_j(s)$ denotes the counting process of type-j events up to time s, and $\mathrm{d}N_j(s)$ represents its infinitesimal increment at time s. The stochastic integral of a function $g(t)$ with respect to N is defined as

$$\int_{-\infty}^{t} g(t)\,\mathrm{d}N(s) := \sum_{j\,:\,t_j \le t} g(t_j), \tag{S13}$$

where $\{t_j\}$ is the set of event timestamps recorded by the counting process $N_j(s)$. Using this definition of the stochastic integral (S13) recovers (S11) from (S12).

We now generalise this formulation. We again consider M different event types, labeled $i = 1, \ldots, M$, where each type corresponds to events occurring on a specific object (for example, a user in a social network or a financial asset). For each type i, the process specifies an intensity function $\lambda_i(t)$ that describes the instantaneous rate at which events of type i are expected to occur at time t. Intuitively, $\lambda_i(t)$ plays the role of a time-varying hazard rate: the probability of observing an event of type i in a short interval $[t, t + dt)$ is equal to $\lambda_i(t), dt$. Formally, the intensity is given by

$$\lambda_i(t) = \underbrace{\mu_i(t)}_{\text{exo (background rate)}} + \underbrace{\sum_{j=1}^{M} \int_{-\infty}^{t} f_{i,j}\;\phi_{i,j}(t - s)\; dN_j(s)}_{\text{endo (self- and cross-excitation)}}\,. \tag{S14}$$

This expression has two parts:

- *Exogenous component.*
  Events may arise independently of prior occurrences, driven by external influences or intrinsic attractiveness. In the context of website traffic, $\mu_i(t)$ captures all forces that direct attention toward page i without relying on internal network dynamics. These forces include media coverage, advertising, social buzz, and the intrinsic appeal or relevance of the content itself. A webpage may therefore attract visits through reputation, topical relevance, or external exposure that amplifies its visibility independently of link-based propagation.
- *Endogenous component.*
  Past events increase the likelihood of future events through self- and cross-excitation mechanisms. An event of type j occurring at time s contributes to the intensity of type i at a later time t through two distinct factors:
  1. The fertility coefficient $f_{i,j}$ specifies the expected number of type-i offspring events directly caused by a single type-j parent event. More precisely, $f_{i,j}$ denotes a stochastic fertility drawn from a probability distribution and represents the average number of type-i events triggered by a type-j event. This construction gives rise to a doubly stochastic process. First, when an event of type j occurs at time s, it is associated with a randomly drawn fertility vector $(f_{i,j})_i$. Second, conditional on this fertility vector, the actual number of daughter events of type i generated by the parent event of type j is random and follows a Poisson distribution with mean $f_{i,j}$. This formulation captures both idiosyncratic event-level heterogeneity, through randomness in fertilities, and stochasticity in event propagation, through Poisson offspring generation.
  2. The kernel $\phi_{i,j}(t-s)$ describes how the influence of a past event decays over time. It models the contribution of events of type j occurring prior to time t to the intensity of type i at time t. We assume an exponential decay of the form $\phi_{i,j}(t) = \frac{1}{\tau_{i,j}} e^{-t/\tau_{i,j}}$, where $\tau_{i,j}$ is a memory parameter. This specification can be generalized to other temporal profiles, such as power-law decay, to capture long-memory effects. The memory kernel $\phi_{i,j}(t-s)$ defines the waiting-time distribution for an event of type i to occur at time t given that an event of type j occurred at time s. Each kernel is normalized such that $\int_0^\infty \phi_{i,j}(u)\,\mathrm{d}u = 1$. For simplicity, and without loss of generality for our main results, we assume that all memory parameters $\tau_{i,j}$ are identical and equal to a common value τ. Under this assumption, the kernel reduces to $\phi_{i,j}(t-s) = \phi(t-s) = \frac{1}{\tau} e^{-(t-s)/\tau}$.

In short, the Hawkes process captures the idea that events can arise both from a background rate of activity and from cascades triggered by earlier events. This allows us to model systems where influence and attention spread dynamically across different objects.

One can define the average number of first-generation daughter events of type i triggered by a mother event of type j,

$$n_{i,j} = \mathbb{E}[f_{i,j}], \tag{S15}$$

where the expectation is taken over all possible draws of the stochastic fertilities. This defines the "branching ratio" from the "mother event" of type j to "daughter events" of type i. The collection of these branching ratios forms the branching ratio matrix

$$N = (n_{i,j}) \ . \tag{S16}$$

Its spectral radius, denoted n, corresponds to the largest eigenvalue of N and determines the stability of the dynamics [12].

To obtain the simplified version presented in the main text, we adopt the following assumption: the fertilities $f_{i,j}$ are no longer random and are fixed at their expectations $n_{i,j}$. This approximation removes randomness in the mothers' fertilities, so that an event of type j generates, on average,

$n_{i,j}$ first-generation events of type i. This simplification yields the multivariate Hawkes process in the form given by expression (S12), which is equivalent to expression (S11), as already mentioned.

S2.2 First Moment of the Hawkes Process

Equation (S11) defines the intensity function $\lambda_i(t)$ of a Hawkes process for process i at time t. We show that the stationary first moment of the Hawkes process recovers Katz centrality (Eq. (S9)) under either of the following two conditions: 1) when the memory parameter tends to zero, or 2) in the asymptotic limit as time approaches infinity.

The Hawkes process is a stochastic point process. Calculating the first moment of the Hawkes process amounts to taking the expectation of expression (S11) with respect to all possible stochastic realizations of the process, which gives:

$$\mathbb{E}[\lambda_i](t) = \mathbb{E}[\mu_i(t)] + \mathbb{E}\left[\sum_{j=1}^{M} \int_{-\infty}^{t} \mathrm{d}N_j(s)\ n_{i,j}\phi\left(t-s\right)\right] \tag{S17a}$$

$$= \mu_i(t) + \sum_{j=1}^{M} \int_{-\infty}^{t} \mathbb{E}[\mathrm{d}N_j(s)] n_{i,j}\phi(t-s) \tag{S17b}$$

$$= \mu_i(t) + \sum_{j=1}^{M} \int_{-\infty}^{t} \mathrm{d}s\ \mathbb{E}[\lambda_j](s) n_{i,j}\phi(t-s). \tag{S17c}$$

S2.2.1 Limit of Infinitely Small Memory Parameter

When the memory parameter τ approaches 0, the function $\phi(t)$ approaches a Dirac functions δ, which gives:

$$\mathbb{E}[\lambda_i](t) = \mu_i(t) + \sum_{j=1}^{M} \int_{-\infty}^{t} \mathrm{d}s\ \mathbb{E}[\lambda_j](s) n_{i,j}\delta(t-s) \tag{S18a}$$

$$= \mu_i(t) + \sum_{j=1}^{M} \mathbb{E}[\lambda_j](t) n_{i,j}. \tag{S18b}$$

By taking $\mu_i = \vec{\beta}$ and $n_{i,j} = \alpha A_{i,j}$, the stationary first moment of the Hawkes process recovers the Katz centrality in Equation (S9). The first moment Hawkes in the univariate case is solution of

$$\mathbb{E}[\lambda](t) = \mu(t) + n\mathbb{E}[\lambda](t), \tag{S19}$$

which yields

$$\mathbb{E}[\lambda](t) = \frac{\mu(t)}{1-n}\ . \tag{S20}$$

As the branching ratio n approaches the critical value 1, the intensity diverges due to runaway self-excitation. The resulting $1/(1-n)$ dependence has a straightforward interpretation: starting from a single mother event, on average it produces n daughters, each of which generates n granddaughters, and so on. The total expected number of events is therefore the geometric series $1+n+n^2+n^3+\cdots = \frac{1}{1-n}$, which diverges as $n \to 1$.

S2.2.2 Asymptotic Long-Time Limit with Constant Exo-Term

Here, we show that the stationary first moment of the Hawkes process recovers the Katz centrality under the condition of taking the long-term time limit.

The Laplace transform of a function $f(x)$ is defined as $\mathcal{L}\{f\}(\beta) = \tilde{f}(\beta) = \int\limits_0^\infty \mathrm{d}t\ f(t)e^{-\beta t}$. Because the Laplace transform of a convolution is given by a multiplication in the Laplace space, the Laplace transform of Equation (S17c) is given by:

$$\mathbb{E}[\tilde{\lambda_i}](\beta) = \tilde{\mu_i}(\beta) + \sum_{j=1}^{M} n_{i,j}\mathbb{E}[\tilde{\lambda_j}(\beta)]\tilde{\phi_{i,j}}(\beta). \tag{S21}$$

For a univariate Hawkes process where the number of event types is $M = 1$, we can immediately solve for the first order moment as:

$$\mathbb{E}[\tilde{\lambda}](\beta) = \tilde{\mu_i}(\beta) + n\mathbb{E}[\tilde{\lambda}(\beta)]\tilde{\phi}(\beta) \tag{S22a}$$

$$= \frac{\tilde{\mu_i}(\beta)}{1 - n\tilde{\phi}(\beta)}. \tag{S22b}$$

The Laplace transform of a constant a is given by $\tilde{a}(\beta) = \frac{a}{\beta}$. When the exogenous intensity is time-invariant, $\mu(t) = \mu$, the Laplace transform of μ is $\tilde{\mu}(\beta) = \frac{\mu}{\beta}$. Substituting in $\tilde{\mu}(\beta)$ gives

$$\mathbb{E}[\tilde{\lambda}](\beta) = \frac{\frac{\mu}{\beta}}{1 - n\tilde{\phi}(\beta)}. \tag{S23}$$

The Laplace transform of the exponential kernel $\phi(t) = \frac{1}{\tau}e^{-\frac{1}{\tau}t}$ is given by

$$\tilde{\phi}(\beta) = \frac{1}{\tau\beta + 1}. \tag{S24}$$

The Taylor expansion of $\tilde{\phi}(\beta)$ in powers of β is given by

$$\tilde{\phi}(\beta) = 1 - \tau\beta + \mathcal{O}(\beta^2). \tag{S25}$$

Because the long-term limit $t \to \infty$ in the time domain corresponds the limit $\beta \to 0$ in the conjugate Laplace domain, after substituting in $\tilde{\phi}(\beta)$, we obtain the stationary long-term solution as,

$$\mathbb{E}[\tilde{\lambda}](\beta) = \frac{\mu}{1-n}\frac{1}{\beta}\left(1 + \frac{n}{1-n}\tau\beta\right)^{-1} = \frac{\mu}{1-n}\frac{1}{\beta}. \tag{S26}$$

This recovers the shape of a constant inverse Laplace transform, and hence by taking the inverse Laplace transform:

$$\mathbb{E}[\lambda] = \frac{\mu}{1-n}, \tag{S27}$$

which recovers Equation (S20).

The derivation can be extended to a multivariate Hawkes process where the number of event types $M > 1$. We denote $Z(\beta) \equiv N\tilde{\phi(\beta)}$, where N is the matrix of branching ratios $n_{i,j}$ defined in equation (S16). We also define

$$\tilde{\Lambda}(\beta) = \begin{pmatrix} \tilde{\lambda_1}(\beta) \\ \vdots \\ \tilde{\lambda_M}(\beta) \end{pmatrix} \qquad \tilde{\mu}(\beta) = \begin{pmatrix} \tilde{\mu_1}(\beta) \\ \vdots \\ \tilde{\mu_M}(\beta) \end{pmatrix}. \tag{S28}$$

Equation (S21) is thus written as:

$$\mathbb{E}[\tilde{\Lambda}](\beta) = \tilde{\mu}(\beta) + \tilde{Z}(\beta)\mathbb{E}[\tilde{\Lambda}](\beta) \tag{S29a}$$
$$= \tilde{\mu}(\beta)(1 - \tilde{Z}(\beta))^{-1}. \tag{S29b}$$

The Taylor expansion of $\tilde{\phi}_{(}\beta)$ as shown in equation (S25) generalizes to vectors in a straightforward way. Substituting in $\tilde{\phi}(\beta)$ obtains

$$\tilde{Z}(\beta) = N(1 - \tau\beta + \mathcal{O}(\beta^2)). \tag{S30}$$

As $\beta \to 0$, $\tilde{Z}(\beta) = N$, and

$$\mathbb{E}[\tilde{\Lambda}](\beta) = [1 - N]^{-1} \begin{pmatrix} \frac{\tilde{\mu}_1}{\beta} \\ \vdots \\ \frac{\tilde{\mu}_M}{\beta} \end{pmatrix}. \tag{S31}$$

The inverse Laplace transform of $\mathbb{E}[\tilde{\Lambda}](\beta)$ is given by

$$\mathbb{E}[\Lambda](t) = [1 - N]^{-1} \begin{pmatrix} \mu_1 \\ \vdots \\ \mu_M \end{pmatrix}, \tag{S32}$$

which is the multivariate generalisation of expression (S20).

This shows that by taking $\mu = \vec{\beta}$ and $n_{i,j} = A_{ij}$, the stationary first moment of the Hawkes process recovers the Katz centrality in Equation (S9) with $\alpha = 1$. Equation (S18b) also recovers the Eigenvector centrality by taking $\mu \to 0$ and $n_{i,j} = \frac{A_{ij}}{n}$. This reveals a key assumption of the Eigenvector centrality that the system is operating at criticality where the intensities are driven purely by endogenous impacts. In addition, Equation (S18b) recovers the PageRank algorithm in Equation (S10) by taking $\mu = \frac{1-d}{N}$ and $n_{i,j} = \frac{d}{L(j)}$. The PageRank algorithm is a simplification of the first moment Hawkes assuming a homogeneous constant exogenous intensity, homogeneity in the branching ratio matrix, and a stationary process with no time dependency.

In the framework of the Hawkes process, the node centrality score can be interpreted as the predicted probability of observable events. We show through the mapping that several centrality measures can be recovered by the first moment Hawkes by making various simplifications, which translates into assumptions imposed on the network systems. This mapping provides an overarching framework to examine and compare different centrality measures and their applicability to social networks.

S2.2.3 Asymptotic Long-Time Limit with Non-Constant Exo-Term

In the previous subsection, the Katz mapping was derived under the assumption that the exogenous term is time-invariant, $\mu_i(t) \equiv \mu_i$. We now consider the case where $\mu(t)$ varies in time.

Taking Laplace transforms of the first-moment equation as in (S21), the mapping from $\mu(t)$ to $\mathbb{E}[\lambda(t)]$ is given in the frequency domain by

$$\mathbb{E}[\tilde{\Lambda}](\beta) = \left(I - N\,\tilde{\phi}(\beta)\right)^{-1} \tilde{\mu}(\beta), \tag{S33}$$

so that $\mu(t)$ is filtered by a matrix-valued transfer function

$$H(\beta) := \left(I - N\,\tilde{\phi}(\beta)\right)^{-1}. \tag{S34}$$

For exponential kernels, $\tilde{\phi}(\beta) = (1+\tau\beta)^{-1}$, and in the low-frequency limit $\beta \to 0$, one has $\tilde{\phi}(\beta) \to 1$, recovering the Katz factor

$$H(\beta) \xrightarrow[\beta\to 0]{} (I-N)^{-1}. \tag{S35}$$

Thus, slow components of $\mu(t)$ (or long-time averages) are amplified exactly as in the Katz mapping.

For finite frequencies, however, the gain becomes frequency-dependent,

$$H(\beta) = \left(I - \frac{N}{1+\tau\beta}\right)^{-1} \tag{S36}$$

which acts as a matrix-valued low-pass filter: slowly varying components of $\mu(t)$ are amplified, while fast fluctuations are attenuated.

In empirical estimation, assuming $\mu(t)$ to be constant when it is in fact time-varying induces a known bias: bursts in $\mu(t)$ are misattributed to endogeneity, pushing estimates of N toward criticality [13–15] Allowing for time variation in $\mu(t)$ (e.g., day-by-day calibration or EM/state-space methods) mitigates this bias and preserves the endo-exo separation.

S2.3 Renormalized Memory Parameter

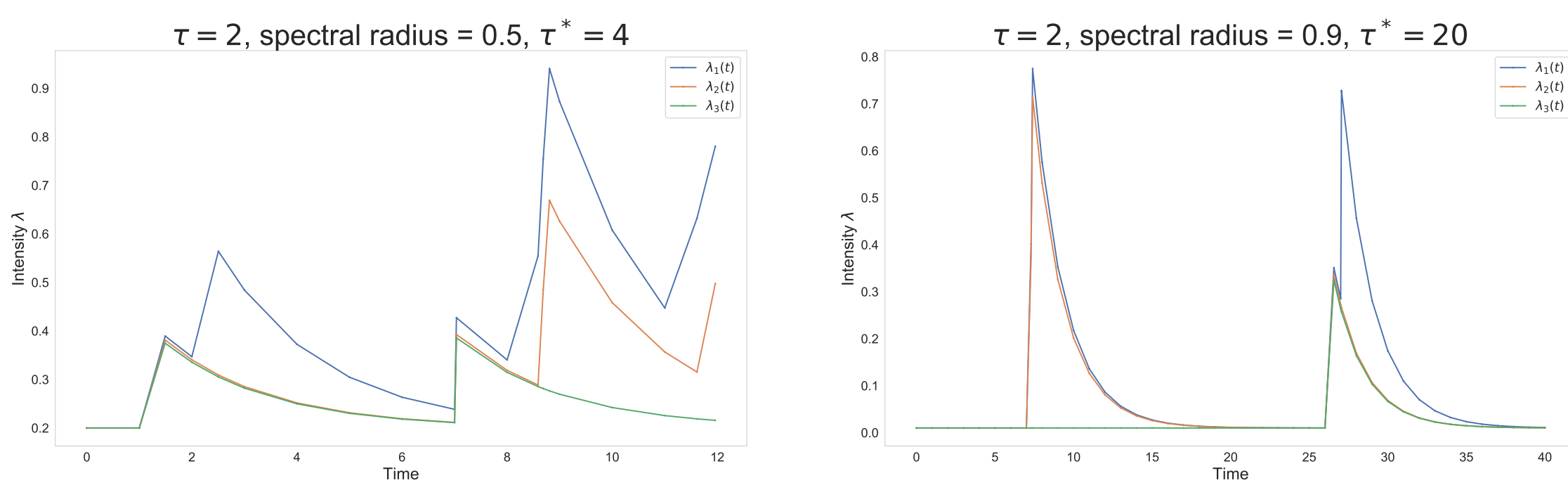

Figure S1: Intensity as a function of observation time of a simulated Hawkes process with $M = 3$ event types, for two different values of the spectral radius of the matrix N of branching ratios $n_{i,j}$ defined in equation (S16). The left plot shows that a memory time τ of 2 is renormalized to 4 for a spectral radius equal to 0.5. The right plot shows that a memory time τ of 2 is renormalized to 20 for a spectral radius equal to 0.9.

The kernel $\phi(t)$ introduces memory into the intensity functions λ in Equation (S11). Building on the previous result that the first moment of the intensity is renormalized by the factor $1/(1-n)$ (in the case $M = 1$, and see expression (S32) for $M > 1$) as the spectral radius approaches 1, we now show that memory effects are similarly rescaled. Consequently, the characteristic fluctuation time scale of λ is of order $\frac{\tau}{1-n}$, as illustrated in Figure S1. This result can be derived as follows (see [16–18] for previous derivations of the renormalization of memory kernels in Hawkes processes in other contexts).

Consider a univariate Hawkes process with baseline (exogenous) intensity $\mu(t)$ as a Dirac function $\delta(t)$ corresponding to a single source event, such that the Laplace transform of $\delta(t)$ is $\delta(\beta) = 1$. The Laplace transform of such a univariate Hawkes process is given by

$$\tilde{\lambda}(\beta) = \tilde{\mu}(\beta) + n\tilde{\lambda}(\beta)\tilde{\phi}(\beta) = \frac{\tilde{\mu}(\beta)}{1-n\tilde{\phi}(\beta)} = \frac{1}{1-n\tilde{\phi}(\beta)}. \tag{S37}$$

The Laplace transform of an exponential memory kernel $\phi(t) = \frac{1}{\tau}e^{-\frac{1}{\tau}t}$ is given by $\tilde{\phi}(\beta) = \frac{1}{\tau\beta+1}$. Substituting for $\tilde{\phi}(\beta)$ in (S37) gives

$$\tilde{\lambda}(\beta) = \frac{1}{1-n(\frac{1}{\tau\beta+1})} = \frac{\tau\beta+1}{1-n+\beta\tau} = \frac{1}{1-n}\,\frac{\tau\beta+1}{1+\frac{\beta\tau}{1-n}}. \tag{S38}$$

Since $\frac{\tau\beta+1}{1-n+\beta\tau} + \frac{n}{1-n+\beta\tau} = 1$, we can rearrange the above equation as

$$\tilde{\lambda}(\beta) = 1 + \frac{n}{1-n}\frac{1}{1+\frac{\tau}{1-n}\beta}. \tag{S39}$$

The inverse Laplace transform of $\tilde{\lambda}(\beta)$ is then given by:

$$\lambda(t) = \delta(t) + \frac{n}{\tau}e^{-\frac{1-n}{\tau}t}\ . \tag{S40}$$

The typical relaxation time is thus renormalized from the bare time scale τ of the bare memory kernel $\phi(t)$ to the renormalized value $\tau^* = \frac{\tau}{1-n}$ by the cascade of self-excitations.

Figure S1 illustrates the renormalization of the effective memory parameter in a three-dimensional Hawkes process simulation. In the left panel, the branching ratio matrix N has spectral radius 0.5 and the baseline memory parameter is $\tau = 2$, which yields a renormalized value $\tau^* = \frac{2}{1-0.5} = 4$. In the right panel, with spectral radius 0.9 and the same baseline $\tau = 2$, the renormalized memory parameter increases dramatically to $\tau^* = \frac{2}{1-0.9} = 20$, clearly visible as a much slower decay of intensity following a peak of activity.

The renormalization of the effective time scale has profound implications for understanding system dynamics. As the branching ratio approaches criticality, interactions retain influence over increasingly long horizons, meaning that the relevant time span for observing, modeling, and interpreting the system is itself state-dependent. This calls for adaptive evaluation methods that adjust to the effective memory of the system rather than relying on fixed observation windows. Ignoring this effect risks truncating long-range dependencies, thereby producing incomplete observations, underestimating causal links, and introducing significant biases in the reconstruction and analysis of networks.

Throughout the manuscript, we express time in units of this renormalized, effective time unit $\tau^* = \frac{\tau}{1-n}$.

S3 Temporal-Network Sensitivity Test

The simulation study in the main text compares HawkesRank with static centrality benchmarks. As an additional sensitivity analysis, we also compare against two temporal-network ranking methods: the dynamic Montegi-Masuda win–loss ranking [19] and tie-decay PageRank [20]. These methods are naturally formulated for time-stamped dyadic interactions, such as games between competitors or explicit user-to-user interactions in online systems. By contrast, the multivariate Hawkes simulations considered here generate node-level event sequences, where events are associated with node types but parent interactions are not directly observed. To apply temporal-network benchmarks in this setting, we therefore construct an inferred temporal edge stream using Hawkes responsibility weights.

For the k-th event of node type i, occurring at time t^i_k, the Hawkes intensity in Eq. (S11) can be decomposed into a baseline contribution and excitation contributions from past events of all node

types:

$$\lambda_i(t_k^i) = \mu_i(t_k^i) + \sum_{j=1}^{M} \sum_{m:t_m^j<t_k^i} n_{i,j} \, \frac{1}{\tau} \exp\left[-\frac{t_k^i - t_m^j}{\tau}\right]. \tag{S41}$$

Equivalently, defining $\mathcal{H}_j(t) = \{t_m^j : t_m^j < t\}$, this can be written as

$$\lambda_i(t_k^i) = \mu_i(t_k^i) + \sum_{j=1}^{M} \sum_{s\in\mathcal{H}_j(t_k^i)} n_{i,j} \, \frac{1}{\tau} \exp\left[-\frac{t_k^i - s}{\tau}\right]. \tag{S42}$$

We then define the aggregate responsibility of parent node type j for the event (i, t_k^i) as

$$r_{j\to i}(t_k^i) = \frac{\sum_{m:t_m^j<t_k^i} n_{i,j} \, \frac{1}{\tau} \exp\left[-\frac{t_k^i - t_m^j}{\tau}\right]}{\lambda_i(t_k^i)}. \tag{S43}$$

Equivalently,

$$r_{j\to i}(t_k^i) = \frac{\sum_{s\in\mathcal{H}_j(t_k^i)} n_{i,j} \, \frac{1}{\tau} \exp\left[-\frac{t_k^i - s}{\tau}\right]}{\lambda_i(t_k^i)}. \tag{S44}$$

Each node-level Hawkes event is therefore converted into a set of weighted fractional temporal edges $j \to i$, with weights $r_{j\to i}(t_k^i)$. This construction avoids assigning equal credit to all potential parent node types while preserving the soft attribution structure implied by the Hawkes process. The remaining fraction,

$$r_{0\to i}(t_k^i) = \frac{\mu_i(t_k^i)}{\lambda_i(t_k^i)},$$

corresponds to the exogenous responsibility, so that

$$r_{0\to i}(t_k^i) + \sum_{j=1}^{M} r_{j\to i}(t_k^i) = 1.$$

The Montegi-Masuda win–loss ranking is computed on the full soft-responsibility edge stream, i.e., using weighted fractional edges rather than binary parent assignments. The method updates win and loss scores recursively, down-weighting older interactions through exponential forgetting while allowing recent interactions to propagate through the network via a transitivity parameter. We use $\alpha_{\mathrm{MM}} = 0.1$ for transitivity propagation and $\beta_{\mathrm{MM}} = 0.05$ for exponential forgetting, distinct from the Katz damping parameter α and exogenous vector β used in the main text.

Tie-decay PageRank is computed by maintaining a time-varying tie matrix $B(t)$. Following the tie-decay recursion, existing ties are first decayed between consecutive event times, after which the current weighted temporal edge contribution is added to the tie matrix. PageRank is then evaluated on the resulting tie-decay network at each time point using damping parameter $d = 0.85$ and tie half-life $\eta_{1/2} = 3$ time units. Because the soft-responsibility construction may generate many weak fractional edges, we use a sparse-responsibility variant for tie-decay PageRank, retaining only parent responsibilities larger than 0.10.

Figure S2 shows the resulting comparison on the same shocked simulation analyzed in Fig. 2 of the main text. The temporal-network benchmarks exhibit qualitatively similar sensitivity to the nonstationary dynamics induced by the exogenous shock and do not systematically improve alignment with the evolving Hawkes intensities relative to the static baselines in this setting.

At the same time, this comparison highlights an important modeling distinction. Temporal-network methods are naturally suited for systems in which explicit dyadic interaction data are directly observed, since they operate on temporal edge streams and can leverage detailed interaction structure. By contrast, HawkesRank is defined directly on node-level event intensities and therefore remains applicable even when only time-stamped node activity is available. In such settings, temporal-network approaches require an additional edge-construction or parent-inference procedure before ranking can be applied, whereas HawkesRank operates directly on the event process itself without requiring an intermediate network reconstruction step.

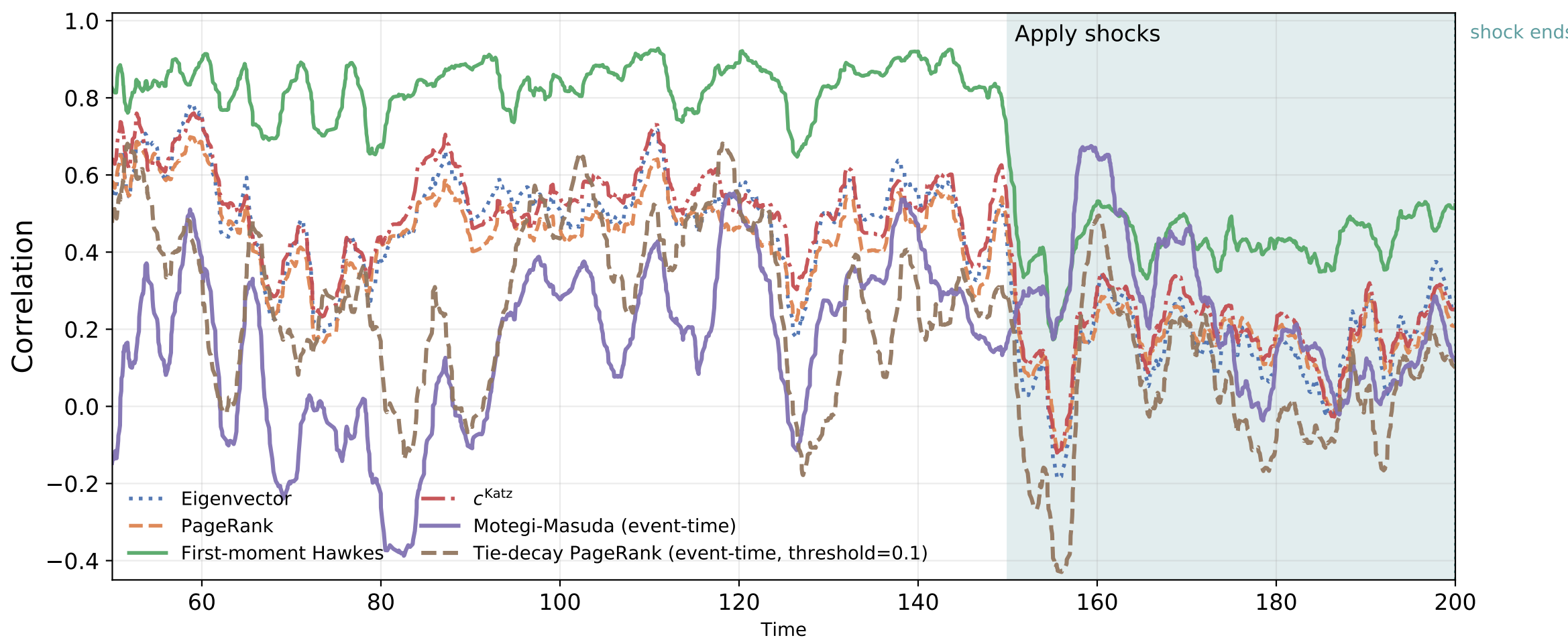

Figure S2: Temporal-network sensitivity analysis for the shocked Hawkes simulation. The static benchmark curves correspond to those reported in Fig. 2 of the main text, and the figure shows the same representative realization. The Motegi–Masuda ranking is computed from the full soft-responsibility temporal edge stream. Tie-decay PageRank is computed from a sparse-responsibility stream retaining only parent responsibilities larger than 0.10. Curves are smoothed as in Fig. 2 of the main text (centered moving average over 30 consecutive points of the intensity evaluation grid); the figure is truncated at $t = 200$, the end of the shock window.

S4 Ensemble Robustness of the Simulation Study

Figure 2 of the main text reports one representative realization of the shocked SEMHP simulation. To verify that the conclusions do not depend on the particular stochastic realization, we repeat the identical experiment over $R = 100$ independent realizations: the network, the baseline vector $\boldsymbol{\mu}$, and the shock schedule are held fixed, and only the random seed of the event-generation process is varied. For each realization we compute, at every point of the intensity evaluation grid, the Spearman rank correlation between the instantaneous ground-truth ranking and each static centrality ranking, and we then aggregate across realizations.

Figure S3 shows the ensemble mean correlation curves together with pointwise 5–95% quantile bands across the 100 realizations. The relative performance reported in the main text is stable across realizations. Averaged over the displayed pre-shock window ($50 \le t < 150$), the first-moment Hawkes ranking attains a Spearman correlation of 0.84 ± 0.02 (mean $\pm$ standard deviation across realizations), compared with 0.56 ± 0.03 for c^{Katz}, 0.55 ± 0.03 for eigenvector centrality, and 0.51 ± 0.03 for PageRank. During the shock window ($150 \le t < 200$), the correlations drop to 0.43 ± 0.02, 0.18 ± 0.03, 0.15 ± 0.03, and 0.16 ± 0.03, respectively. The ordering of the methods is

deterministic in practice: the first-moment Hawkes ranking achieves the highest correlation among the static benchmarks in 100% of the realizations, both before and during the shock, and every static method exhibits a pronounced drop in correlation upon shock onset in 100% of the realizations. The representative realization shown in Fig. 2 of the main text is typical: its phase-averaged correlations lie well within the ensemble bands.

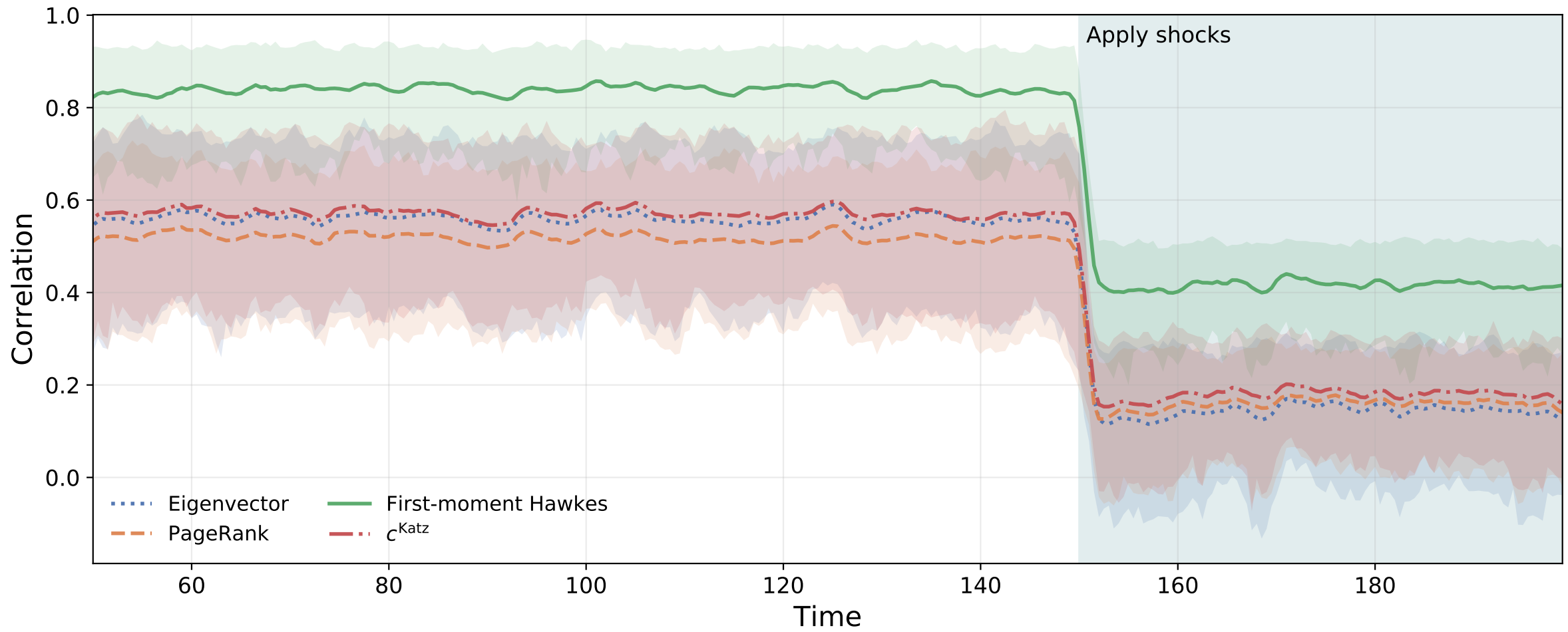

Figure S3: Ensemble robustness of the simulation study of Fig. 2 of the main text. Lines show the mean Spearman correlation between the instantaneous ground-truth ranking and each static centrality ranking, averaged over $R = 100$ independent realizations of the SEMHP simulation with identical parameters (network, baseline intensities, and shock schedule held fixed; only the simulation seed varies). Shaded bands indicate the pointwise 5–95% quantile range across realizations. Each realization's correlation curve is smoothed exactly as in Fig. 2 of the main text (centered moving average over 30 consecutive points of the intensity evaluation grid) before aggregation. The shaded vertical region marks the exogenous shock interval ($150 \leq t < 200$), during which the smallest baseline intensity is increased ten-fold.

S5 Network Dependency on Construction Parameters

The lead–lag correlation-based emotion network described in the main text depends on two parameters: the bin size b, which determines the temporal aggregation window used to construct emotion activity time series, and the lag parameter ℓ, which specifies how many bins separate the two observations when evaluating lead–lag correlations. Together, these parameters determine how the underlying event data are discretized and how temporal precedence between emotions is measured.

To illustrate the sensitivity of the resulting networks to these choices, Fig. S4 shows adjacency matrices constructed from the same chat data using different combinations of b and ℓ. Although the underlying sequence of messages is identical, the inferred correlations between emotions vary substantially across parameter settings, producing markedly different interaction patterns. This demonstrates a general limitation of correlation-based network constructions: the inferred adjacency structure can depend strongly on arbitrary modeling choices.

S6 Out-of-Sample Predictive Validation

The primary objective of HawkesRank is to provide a dynamic ranking framework rather than a predictive model. Nevertheless, it is informative to assess its out-of-sample predictive value relative

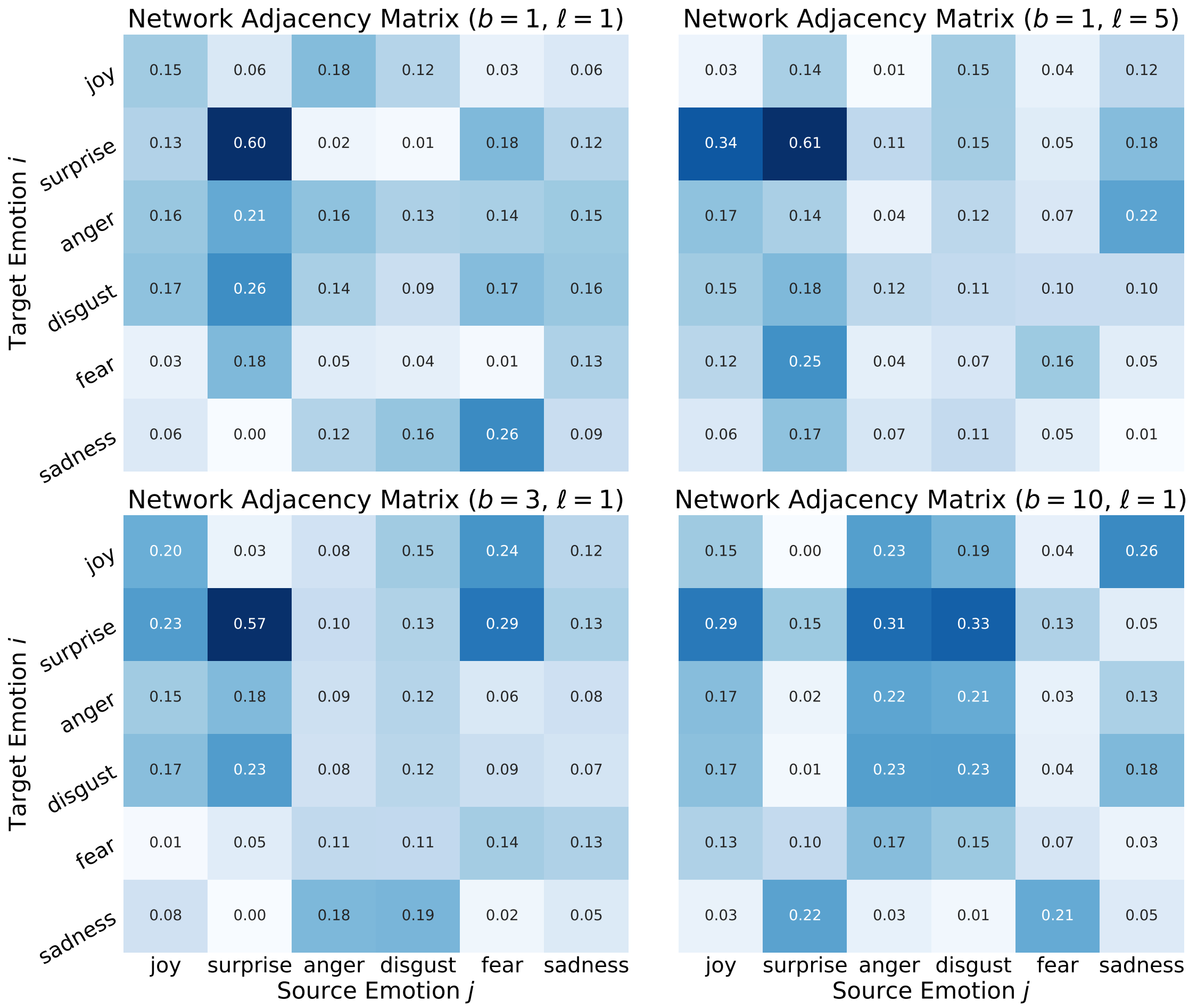

Figure S4: Adjacency matrices of correlation-based emotion networks constructed using different combinations of bin size b and lag parameter ℓ. The bin size determines the temporal aggregation window used to construct emotion activity time series, while the lag parameter specifies the number of bins separating the two observations when computing lead–lag correlations. Each panel is computed from the same YouTube live-chat data using different parameter values (b, ℓ).

to the more traditional lead–lag correlation-network representation. To this end, each of the 397 YouTube Live chat sequences is split chronologically. The first 70% of each observation window is used for estimation, and the remaining 30% is reserved for out-of-sample evaluation. This yields 74,960 training events and 31,269 held-out events.

On the training data, we re-estimate the Hawkes model as described in Ref. [21]. The estimated components include the branching matrix, the transcript-driven exogenous intensities, and the memory parameters. We use the same model class, parameter bounds, likelihood function, and optimization procedure as in the analysis from the main text.

Immediately before each held-out message at time t, the Hawkes model assigns probability

$$p_i(t) = \frac{\lambda_i(t)}{\sum_{m=1}^{M} \lambda_m(t)}$$

to emotion i. The intensities are evaluated using only the event history observed up to time t^-, while all model parameters remain fixed at their training-sample estimates.

For comparison, the lead–lag correlation network is estimated separately on each video's training window. Chat messages are aggregated into bins of width $b = 0.5$ minutes, and the adjacency entry $A_{i,j}$ is defined as the lagged correlation between the activity of emotion j and the subsequent activity of emotion i at lag $\ell = 2$. At prediction time, the most recent lagged emotion-count vector is propagated through the estimated adjacency matrix. The resulting scores are normalized to obtain a probability distribution over the six emotions. We additionally include uniform guessing as a chance-level reference.

Predictive performance is evaluated using cross-entropy, defined as the average negative natural logarithm of the probability assigned to the emotion that actually occurs. Lower values therefore indicate better probabilistic predictions. Because cross-entropy uses the full predicted probability distribution, it remains informative even when one class dominates the data. This is important here because *joy* accounts for approximately 76% of all emotion events, making top-1 accuracy largely insensitive to differences between the competing methods.

Table S1 summarizes the out-of-sample results. Uniform guessing attains the expected cross-entropy of $\ln(6) \approx 1.79$. The lead–lag correlation network achieves a cross-entropy of 1.71 ± 0.02 and therefore performs only marginally better than chance. HawkesRank achieves a substantially lower cross-entropy of 0.86 ± 0.01.

The difference in cross-entropy also admits a direct probabilistic interpretation. Let p_k denote the probability that a model assigns to the emotion that actually occurs for held-out message k, where $k = 1, \ldots, K$ enumerates all held-out messages across all videos. The average cross-entropy is then $\mathrm{CE} = -\frac{1}{K}\sum_{k=1}^{K} \ln p_k$. Using the identity $\exp(-\mathrm{CE}) = \left(\prod_{k=1}^{K} p_k\right)^{1/K}$, we see that the exponential of the negative cross-entropy is simply the geometric mean probability assigned to the emotion that actually occurs. Consequently, the difference between two cross-entropies directly measures the ratio of these typical assigned probabilities. In the present case, $\exp(1.71 - 0.86) \approx 2.3$, meaning that HawkesRank assigns, on average, approximately 2.3 times higher probability to the emotion that occurs next than the lead–lag correlation network.

Table S1: Out-of-sample predictive performance on the held-out YouTube Live chat data. Lower cross-entropy indicates better probabilistic prediction. Uncertainty values denote bootstrap standard errors obtained by resampling videos.

Method	Average cross-entropy
Uniform guessing	1.79
Lead–lag correlation network	1.71 ± 0.02
HawkesRank	0.86 ± 0.01

References

[1] Sergey Brin and Lawrence Page. The anatomy of a large-scale hypertextual web search engine. *Computer Networks and ISDN Systems*, 30(1-7):107–117, 1998.

[2] David F Gleich. Pagerank beyond the web. *SIAM Review*, 57(3):321–363, 2015.

[3] Phillip Bonacich. Power and centrality: A family of measures. *American Journal of Sociology*, 92(5):1170–1182, 1987.

[4] Polina Rozenshtein, Nikolaj Tatti, and Aristides Gionis. Finding events in temporal networks: Segmentation meets densest subgraph discovery. In *2016 IEEE 16th International Conference on Data Mining (ICDM)*, pages 547–556. IEEE, 2016.

[5] Myunghwan Kim and Ross Anderson. Temporal node centrality in complex networks. *Physical Review E*, 85(2):026107, 2012.

[6] Peter Grindrod, Desmond J Higham, Mark C Parsons, and Ernesto Estrada. Communicability across evolving networks. *Physical Review E*, 83(4):046120, 2011.

[7] Matheus R. F. Mendonça, André M. S. Barreto, and Artur Ziviani. Approximating network centrality measures using node embedding and machine learning. *IEEE Transactions on Network Science and Engineering*, 8(1):220–230, 2021.

[8] Peter Grindrod and Desmond J Higham. A dynamical systems view of network centrality. *Proc. R. Soc. A*, 470:20130835, 2014.

[9] Felipe Grando, Lisandro Zambenedetti Granville, and Luis C. Lamb. Machine learning in network centrality measures: Tutorial and outlook. *ACM Computing Surveys (CSUR)*, 51(5): 1–32, 2018.

[10] Xin Li, Liav Bachar, and Rami Puzis. Centrality learning: Auralization and route fitting. *Entropy*, 25:1115, 2023.

[11] Mateusz Stolarski, Adam Piróg, and Piotr Bródka. Identifying key nodes for influence spread using machine learning. *Entropy*, 26(11):955, 2024.

[12] D.J. Daley and D. Vere-Jones. *An Introduction to the Theory of Point Processes*. Vol. I & II, Springer, 2008.

[13] Vladimir Filimonov and Didier Sornette. Apparent criticality and calibration issues in the hawkes self-excited point process model: application to high-frequency financial data. *Quantitative Finance*, 15:11293–1314, 2015.

[14] Spencer Wheatley, Alexander Wehrli, and Didier Sornette. The endo–exo problem in high frequency financial price fluctuations and rejecting criticality. *Quantitative Finance*, 19(7): 1165–1178, 2019.

[15] Alexander Wehrli, Spencer Wheatley, and Didier Sornette. Scale-, time-and asset-dependence of Hawkes process estimates on high frequency price changes. *Quantitative Finance*, 21(5): 729–752, 2021.

[16] A. Sornette and D. Sornette. Renormalization of earthquake aftershocks. *Geophysical Research Letters*, 26(13):1981–1984, 1999.

[17] A. Helmstetter and D. Sornette. Subcritical and supercritical regimes in epidemic models of earthquake aftershocks. *Journal of Geophysical Research: Solid Earth*, 107(B10):2237, doi:10.1029/2001JB001580, 2002.

[18] Qinghua Lei and Didier Sornette. Endo-exo framework for a unifying classification of episodic landslide movements: Implications for forecasting catastrophic failures. *Science Advances*, 11 (39):eady9141, 2025.

[19] Shun Motegi and Naoki Masuda. A network-based dynamical ranking system for competitive sports. *Scientific reports*, 2(1):904, 2012.

[20] Walid Ahmad, Mason A Porter, and Mariano Beguerisse-Díaz. Tie-decay networks in continuous time and eigenvector-based centralities. *IEEE Transactions on Network Science and Engineering*, 8(2):1759–1771, 2021.

[21] Yishan Luo, Didier Sornette, and Sandro Claudio Lera. Social feedback amplifies emotional language in online video live chats. *Communications Psychology*, 3(1):188, 2025. ISSN 2731-9121.
